# Characteristics of fatigue in Parkinson’s disease: A longitudinal cohort study

**DOI:** 10.3389/fnagi.2023.1133705

**Published:** 2023-03-10

**Authors:** Xiaoxia Zhou, Yaqin Xiang, Tingwei Song, Yuwen Zhao, Hongxu Pan, Qian Xu, Yase Chen, Qiying Sun, Xinyin Wu, Xinxiang Yan, Jifeng Guo, Beisha Tang, Lifang Lei, Zhenhua Liu, for Parkinson's Disease & Movement Disorders Multicenter Database and Collaborative Network in China (PD-MDCNC)

**Affiliations:** ^1^Department of Neurology, Xiangya Hospital, Central South University, Changsha, China; ^2^Department of Geriatrics, Xiangya Hospital, Central South University, Changsha, China; ^3^National Clinical Research Center for Geriatric Disorders, Xiangya Hospital, Central South University, Changsha, China; ^4^Department of Epidemiology and Health Statistics, Xiangya School of Public Health, Central South University, Changsha, China; ^5^Key Laboratory of Hunan Province in Neurodegenerative Disorders, Central South University, Changsha, China; ^6^Department of Neurology, The Third Xiangya Hospital, Central South University, Changsha, China

**Keywords:** Parkinson’s disease, fatigue, longitudinal, PD-MDCNC, progression

## Abstract

**Objective:**

To assess the prevalence, evolution, clinical characteristics, correlates and predictors of fatigue as well as to investigate the influence of comorbid fatigue on the longitudinal changes in motor and non-motor symptoms over a 2-year longitudinal follow-up period in a large cohort of patients with Parkinson’s disease (PD).

**Materials and methods:**

A total of 2,100 PD patients were enrolled from the Parkinson’s Disease & Movement Disorders Multicenter Database and Collaborative Network in China (PD-MDCNC), and their motor and non-motor symptoms were assessed biennially using comprehensive scales, including the 16-item Parkinson Fatigue Scale (PFS-16). Each PD patient was categorized as PD with or without fatigue on the basis of a cut-off mean PFS-16 score of 3.3.

**Results:**

The prevalence of fatigue in our cohort was 36.8%. Compared to PD patients without fatigue, PD patients with fatigue were more likely to be older, have a longer disease duration, and higher baseline levodopa equivalent daily dose (all *p* < 0.05). Moreover, PD patients with fatigue showed more severe motor and non-motor phenotypes than those without fatigue. Overall, high total Unified Parkinson’s Disease Rating Scale (UPDRS) score (odds ratio [OR] = 1.016, 95% confidence interval [CI]: 1.009–1.024), Non-Motor Symptoms Scale score (OR = 1.022, 95% CI: 1.015–1.029), postural instability and gait difficulty (PIGD) subtype (OR = 1.586, 95% CI: 1.211–2.079), presence of excessive daytime sleepiness (EDS; OR = 1.343, 95% CI: 1.083–1.666), and wearing-off (OR = 1.282, 95% CI: 1.023–1.607) were significantly associated with fatigue in PD patients (all *p* < 0.05). High total UPDRS score at baseline (OR = 1.014, 95% CI: 1.002–1.027, *p* = 0.028) increased the risk of developing fatigue during follow-up. Although significant, the odds ratios were low and confidence intervals were narrow. Analysis of disease progression showed significant group differences in motor and non-motor symptoms. In comparison with the never-fatigue group, the persistent-fatigue group showed significantly greater progression in motor, autonomic dysfunction, sleep, depression and cognitive symptoms (all *p* < 0.05).

**Conclusion:**

Increased disease severity, presence of the PIGD subtype, EDS, and wearing-off were associated with fatigue in PD patients. Significant subgroup-level differences were observed in the progression of motor and non-motor symptoms across different fatigue subgroups of PD patients.

## 1. Introduction

Parkinson’s disease (PD) is the most common movement disorder and second-most common neurodegenerative disorder in the world after Alzheimer’s disease, affecting >1% of the population aged ≥65 years and expected to double in prevalence by 2030 (Aarsland et al., 2021). PD is pathologically characterized by dopaminergic neuronal loss in the substantia nigra pars compacta and the presence of intracellular inclusions containing α-synuclein aggregates (Armstrong and Okun, 2020; Liu et al., 2022). Considering that the progression of PD can span decades, it has adverse and profound consequences for patients, caregivers, and the society (Bloem et al., 2021).

Apart from cardinal motor features, such as bradykinesia (slowness of movement), rigidity, and resting tremor, PD is also associated with a heterogeneous spectrum of non-motor symptoms that contribute greatly to the overall disease burden (Schapira et al., 2017; Aarsland et al., 2021). Notably, fatigue is a common but less visible symptom of PD and affects approximately half of all patients (Kluger, 2017), which is often considered by patients with PD to be one of the most disabling symptoms affecting their daily activities and quality of life (Stocchi et al., 2014). As demonstrated by the growing number of research articles, interest in the subject is increasing. Furthermore, expert opinions on raising awareness on fatigue have been published (Lazcano-Ocampo et al., 2020). Considering that fatigue has diverse clinical manifestation, large clinical heterogeneity with many confounding factors, and lacks of clear pathogenesis and treatment to date, the understanding of fatigue in PD is insufficient. Although previous studies on fatigue in PD have mostly focused on the clinical features and factors associated with PD, fatigue has also been shown to be associated with other common non-motor symptoms of PD, including cognitive impairment, depression, apathy, anxiety, autonomic dysfunction, daytime somnolence, and sleep disturbances, which adversely affects patients’ quality of life (Stocchi et al., 2014; Fu et al., 2016; Kluger et al., 2017; Siciliano et al., 2018; Ongre et al., 2021; Ou et al., 2021; Zhou et al., 2021; Béreau et al., 2022). However, the results of these studies are not always consistent. For example, Ongre et al. reported that a higher level of fatigue was associated with better cognitive functioning, whereas Kluger et al. and Siciliano et al. found that fatigue is associated with cognitive decline. Moreover, most published investigations were limited in size (sample size <500), and knowledge regarding the longitudinal progression of fatigue (Ongre et al., 2021; Ou et al., 2021) in PD is currently limited. Furthermore, the potential influence of the presence of concomitant fatigue in PD on the rate of progression of other phenotypic features has not been characterized.

In the light of these discrepancies and gaps in the literature, we conducted a longitudinal cohort study of patients with PD with a 2-year follow-up period. The aims of this study were to assess the prevalence and evolution, clinical characteristics, correlates and predictors of fatigue, as well as to investigate the influence of comorbid fatigue on the longitudinal changes in motor and non-motor symptoms in a large cohort of patients with PD.

## 2. Materials and methods

### 2.1. Participants

The participants were enrolled from the Parkinson’s Disease & Movement Disorders Multicenter Database and Collaborative Network in China (PD-MDCNC) between January 2017 and August 2022. At least two neurological specialists confirmed the clinical diagnosis of PD according to the Movement Disorder Society Clinical Diagnostic Criteria for Parkinson’s Disease (Postuma et al., 2015), including diagnoses of either clinically established or probable PD. The exclusion criteria were as follows: (1) failure to complete the 16-item Parkinson’s Fatigue Scale (PFS-16) questionnaire at baseline and follow-up, (2) missing data ≥10%, or (3) diagnosis of other causes of parkinsonism on baseline or follow-up assessments. The clinical data of all participants were stored in the PD-MDCNC.^1^ Written informed consent was obtained from all participants. This study was approved by the Ethics Committee of Xiangya Hospital and conducted in accordance with the ethical guidelines of the Declaration of Helsinki.

### 2.2. Clinical assessments

The participants underwent comprehensive and standardized clinical assessments at baseline and at the 2-year follow-up. Before conducting the assessments, all researchers were trained to ensure equal understanding of the scales as well as the methods and phrasing used for clinical data collection. Baseline clinical information, including age, sex, age at onset, and disease duration, was collected. Treatments were recorded during the baseline and 2-year follow-up interviews. The levodopa equivalent daily dose (LEDD) was calculated using a commonly used method (Tomlinson et al., 2010). Clinical examinations of motor symptoms and a broad range of non-motor symptoms were performed on the basis of a series of PD assessment scales. Motor symptoms were assessed based on the Unified Parkinson’s Disease Rating Scale (UPDRS) and Hoehn and Yahr stages, defining motor subtypes as tremor dominant (TD), postural instability and gait difficulty (PIGD), or indeterminate. The ratio of the mean UPDRS tremor score (8 items) to the mean UPDRS PIGD score (5 items) was used to define the TD (ratio ≥ 1.5), PIGD (ratio ≤ 1), and indeterminate subtypes (ratio > 1.0 and < 1.5; Jankovic et al., 1990; Stebbins et al., 2013). Motor complications, such as dyskinesia and wearing-off were diagnosed by clinicians, and the severities of dyskinesia and wearing-off were evaluated by UPDRS part IV-A and 9-item Wearing-off Questionnaire (WOQ-9), respectively. Freezing of gait (FOG) was evaluated using the New Freezing of Gait Questionnaire (NFOGQ).

In addition to motor symptoms, we evaluated a broad range of non-motor symptoms based on the Non-Motor Symptoms Scale (NMSS), Scales for outcomes in Parkinson’s disease-Autonomic Dysfunction (SCOPA-AUT), Mini-Mental State Examination (MMSE), Rapid Eye Movement Sleep Behavior Disorder Questionnaire-Hong Kong (RBDQ-HK), Epworth Sleepiness Scale (ESS), Parkinson’s Disease Sleep Scale (PDSS), Hyposmia Rating Scale (HRS), Functional Constipation Diagnostic Criteria Rome III, and Hamilton Depression Rating Scale (HAMD-17). Quality of life was assessed using the Parkinson’s disease questionnaire (PDQ-39). Patients with illiteracy, primary education, and above junior education were identified as having cognitive impairment when the MMSE scores were below 17, 20 and 24 points, respectively. Hyposmia was defined as a total HRS score less than 22.5. Probable rapid eye movement sleep behavior disorder (pRBD) was defined as a total RBDQ-HK scale score no less than 18. Excessive daytime sleepiness (EDS) was defined as a total ESS score higher than 10. Depression was defined as a total HAMD-17 score higher than 7. Details regarding these clinical scales have been provided in our previous study (Zhao et al., 2020; Deng et al., 2022; Zhou X. et al., 2022; Zhou Z. et al., 2022).

### 2.3. Definition of fatigue

Fatigue was assessed using PFS-16, which has been developed for use in routine clinical practice and recommended for screening and rating the severity of fatigue. The PFS is a 16-item patient-rated scale that encompasses the physical aspects of fatigue and its impact on patients’ daily functioning. Item scores ranged from 1 (strongly disagree) to 5 (strongly agree), with the PFS-16 mean score calculated as the mean of all individual item scores (range: 1.0–5.0). Based on a previous study (Brown et al., 2005; Friedman et al., 2010), we used a threshold PFS-16 mean score of 3.3 to define the presence of fatigue in our study. Considering that fatigue was not necessarily persistent, we classified fatigue into the following four types (Alves et al., 2004): “never fatigue” referred to cases showing no fatigue at baseline and the 2-year follow-up; “non-persistent fatigue” indicated fatigue that disappeared during the 2-year follow-up; “new-onset fatigue” indicated fatigue that first appeared at the 2-year follow-up; and “persistent fatigue” indicated fatigue that appeared at both the baseline and 2-year follow-up.

### 2.4. Statistical analyses

Continuous variables were summarized using descriptive statistics, and categorical variables were summarized using patient counts and percentages. For the analysis of cross-sectional data, comparisons between patients with and without fatigue were made using the chi-squared test and multivariable logistic regression for qualitative variables, and Student’s t-test or nonparametric Mann–Whitney test and multivariable linear regression for quantitative variables. Most comparisons were adjusted for age and sex, and for additional variables thought to be potential confounders.

Spearman correlation analysis was used to analyze relationships between the presence of fatigue and other clinical variables. Binary logistic regression models were used to assess the factors associated with fatigue in PD and included as many variables as possible. A binary dependent variable of fatigue was assigned a value of 0 when the PFS-16 mean score was <3.3 and a value of 1 when the score was ≥3.3. The following covariates were included in the logistic model after analysis of multicollinearity (variance inflation factor < 10): age, sex, body mass index (BMI), disease duration, LEDD, total UPDRS score, NMSS score, PDSS score, PDQ-39 score, SCOPA-AUT score, motor subtype, severity of PD (Hoehn and Yahr stages 1–2.5 vs. stages 3–5), constipation (presence vs. absence), cognitive impairment (presence vs. absence), hyposmia (presence vs. absence), pRBD (presence vs. absence), EDS (presence vs. absence), depression (presence vs. absence), FOG (presence vs. absence), wearing-off (presence vs. absence), and dyskinesia (presence vs. absence).

The binary logistic regression model was also used to investigate the clinical predictors for fatigue in PD. The analysis was based on the patients who had no fatigue at baseline. The clinical outcome was the new occurrence of fatigue during follow-up. In the multivariate model, the covariates of the above binary logistic regression analysis after analysis of multicollinearity (variance inflation factor < 10) were included as covariates.

For the analysis of longitudinal data, the outcomes of clinical evaluations were compared using a paired-sample t-test, Wilcoxon matched-pair signed-rank test, or McNemar–Bowker test. We used generalized estimating equation (GEE) for comprehensive longitudinal comparison of the progression of the four fatigue subgroups. In each separate GEE for progression, the clinical characteristics at follow-up were defined as dependent variables. To reduce the “regression toward mean” bias, the analysis was adjusted for age at baseline, sex, disease duration, and baseline values of the clinical factors (Vickers and Altman, 2001). All analyses were performed using SPSS version 25.0 (IBM Corp., Armonk, NY, United States). Two-tailed *p*-values <0.05 were considered the threshold for statistically significant differences in all analyses.

## 3. Results

### 3.1. Overview, prevalence, and evolution of fatigue

Overall, 2,100 patients were eligible for inclusion in the study. The main demographic and clinical characteristics of the patients are summarized in Table 1. At baseline, the mean age, age at onset, and disease duration were 60.47 ± 10.21, 55.06 ± 10.81, and 5.42 ± 4.52 years, respectively. Approximately half of the patients were men (49.9%) and three-quarters of the patients (77.4%) were in Hoehn and Yahr stages 1–2.5. The baseline LEDD was 490.05 ± 278.44 mg (Table 1).

**Table 1:** Demographic and clinical characteristics of patients with PD at baseline.

Characteristics	Overall (*n* = 2,100)	PD with fatigue (*n* = 772)	PD without fatigue (*n* = 1,328)	*p* value^a^	*p* value^b^
Age at baseline	60.47 ± 10.21	61.49 ± 10.00	59.88 ± 10.28	**<0.001**	NA
Age at onset	55.06 ± 10.81	55.31 ± 10.48	54.91 ± 11.00	0.272	NA
Gender ratio (male)	1,047(49.9%)	387(50.1%)	660(49.7%)	0.849	NA
BMI	22.78 ± 5.22	22.59 ± 7.46	22.89 ± 3.27	**<0.001**	0.507
PD duration	5.42 ± 4.52	6.20 ± 4.80	4.97 ± 4.28	**<0.001**	NA
LEDD	490.05 ± 278.44	525.00 ± 280.70	469.73 ± 275.19	**<0.001**	**0.010**
Motor symptoms					
UPDRS total score	39.86 ± 20.40	48.45 ± 20.77	34.86 ± 18.43	**<0.001**	**<0.001**
UPDRS Part III score	25.15 ± 13.97	30.08 ± 14.38	22.29 ± 12.88	**<0.001**	**<0.001**
Bradykinesia score	9.29 ± 6.00	11.09 ± 6.34	8.25 ± 5.53	**<0.001**	**<0.001**
Rigidity score	5.19 ± 4.00	6.38 ± 4.26	4.49 ± 3.67	**<0.001**	**<0.001**
Tremor score	3.33 ± 3.30	3.61 ± 3.73	3.16 ± 3.02	**0.256**	0.140
Postural instability score	3.73 ± 2.76	4.64 ± 2.99	3.20 ± 2.46	**<0.001**	**<0.001**
Motor subtype				**<0.001**	
Tremor-dominant	460(21.9%)	115(14.9%)	345(26.0%)		
Intermediate type	291(13.9%)	95(12.3%)	196(14.8%)		**0.038**
PIGD-dominant	1,349(64.2%)	562(72.8%)	787(59.3%)		**<0.001**
Hoehn and Yahr stages				**<0.001**	**<0.001**
Stages of 1–2.5	1,625(77.4%)	516(66.8%)	1,109(83.5%)		
Stages of 3–5	475(22.6%)	256(33.2%)	219(16.5%)		
Freezing of gait^c^	588(28.0%)	286(37.0%)	302(22.7%)	**<0.001**	**<0.001**
Non-motor symptoms					
NMSS total score	33.96 ± 25.55	46.71 ± 28.67	26.55 ± 20.13	**<0.001**	**<0.001**
PDSS total score	119.28 ± 28.60	110.88 ± 25.33	124.16 ± 29.26	**<0.001**	**<0.001**
PDQ-39 total score	26.09 ± 31.46	36.03 ± 28.81	20.31 ± 31.50	**<0.001**	**<0.001**
SCOPA-AUT score	8.07 ± 5.42	9.61 ± 5.71	7.17 ± 5.04	**<0.001**	**<0.001**
Constipation^d^	760(36.2%)	355(46.0%)	405(30.5%)	**<0.001**	**<0.001**
CI^e^	149(7.1%)	78(10.1%)	71(5.3%)	**<0.001**	**0.002**
MMSE score	26.73 ± 3.37	26.23 ± 3.75	27.03 ± 3.09	**<0.001**	**<0.001**
Hyposmia^f^	899(42.8%)	386(50.0%)	513(38.6%)	**<0.001**	**<0.001**
HRS score	19.58 ± 6.22	18.62 ± 6.60	20.13 ± 5.92	**<0.001**	**<0.001**
pRBD^g^	882(42.0%)	397(51.4%)	485(36.5%)	**<0.001**	**<0.001**
RBDQ-HK score	16.32 ± 16.43	20.23 ± 17.43	14.06 ± 15.38	**<0.001**	**<0.001**
EDS^h^	677(32.2%)	333(43.1%)	344(25.9%)	**<0.001**	**<0.001**
ESS score	7.37 ± 6.16	9.01 ± 6.50	6.41 ± 5.75	**<0.001**	**<0.001**
Depression^i^	685(32.6%)	371(48.1%)	314(23.6%)	**<0.001**	**<0.001**
HAMD score	5.31 ± 5.19	7.35 ± 5.79	4.12 ± 4.38	**<0.001**	**<0.001**
Motor complications					
Wearing-off^j^	757(36.0%)	358(46.4%)	399(30.0%)	**<0.001**	**<0.001**
Dyskinesia^k^	288(13.7%)	138(17.9%)	150(11.3%)	**<0.001**	**0.002**

Data are mean ± SD or *n* (%), unless otherwise indicated. *p* value^a^ are from the chi-squared test, Student’s *t*-test or nonparametric Mann–Whitney test; *p* value^b^ are from multivariable linear or logistic regression (controlled for sex, age, disease duration). Significant *p* values are indicated in bold. ^c–k^Evaluated, respectively, by New Freezing of Gait Questionnaire (NFOGQ), Functional Constipation Diagnostic Criteria Rome III (ROME III), MMSE, HRS, RBDQ-HK, ESS, HAMD, 9-item Wearing-off Questionnaire (WOQ-9) and UPDRS part IV-A. PD, Parkinson’s disease; BMI, body mass index; LEDD, levodopa equivalent daily dose; UPDRS, unified Parkinson’s disease rating scale; PIGD, postural instability and gait difficulty; NMSS, non-motor symptom scale; PDSS, Parkinson’s disease sleep scale; PDQ-39, The Parkinson’s disease questionnaire (PDQ-39); SCOPA-AUT, scales for outcomes in Parkinson’s disease-autonomic dysfunction; CI, cognitive impairment; MMSE, Mini-mental state examination; HRS, Hyposmia rating scale; pRBD, probable rapid eye movement sleep behavior disorder; RBDQ-HK, rapid eye movement sleep Behavior disorder questionnaire-Hong Kong; EDS, excessive daytime sleepiness; ESS, Epworth sleepiness scale; HAMD, Hamilton depression rating scale (HAMD-17).

**Figure 1:**
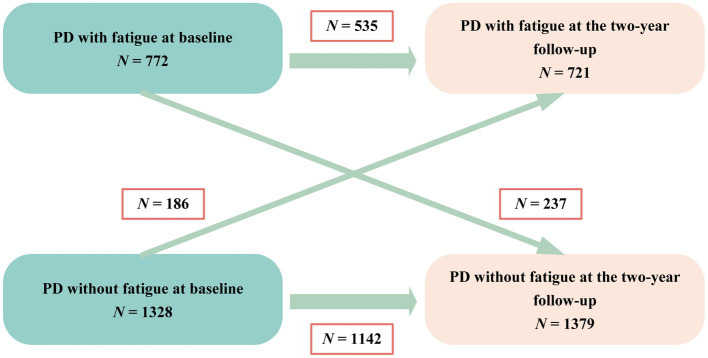
Diagram of fatigue change over time. PD, Parkinson’s disease.

A total of 772 (36.8%) patients were identified as experiencing fatigue on the basis of the baseline PFS-16 score (PFS-16 mean score ≥ 3.3 points). The prevalence of fatigue by disease duration group was 26.4% (disease duration ≤3 years), 41.2% (3 < disease duration < 10 years) and 46.4% (disease duration ≥ 10 years). The frequency of fatigue was 31.7 and 53.9% in patients with Hoehn and Yahr stages 1–2.5 and 3–5, respectively (Supplementary Table 1). Fatigue did not always persist from one visit to the next in every patient during the follow-up period (Figure 1). The results showed never fatigue in 54.4% (1,142/2,100), non-persistent fatigue in 11.3% (237/2,100), new-onset fatigue in 8.8% (186/2,100), and persistent fatigue in 25.5% (535/2,100) of the patients.

To control for the heterogeneity of patient timelines, we stratified PD cases into three subgroups based on disease duration, as shown in Supplementary Table 2. Most scores of the motor and non-motor symptoms, including fatigue, worsened and the prevalence of symptoms increased as the disease progressed. Additionally, we found that motor complications were prone to occur in patients with PD with longer disease duration.

### 3.2. Comparison of clinical characteristics between PD patients with and without fatigue

**Figure 2:**
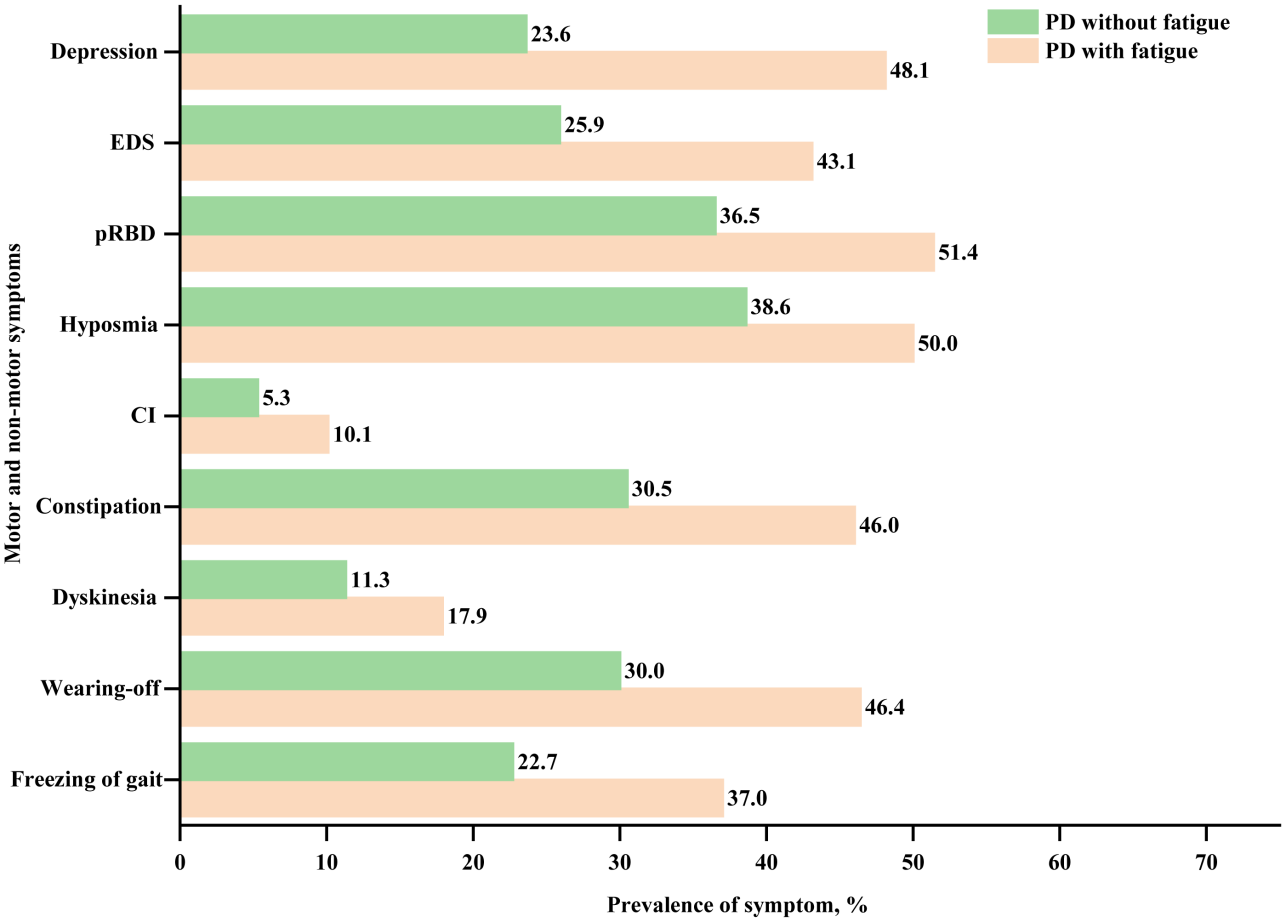
Frequency of motor and non-motor symptoms in PD with and without fatigue at baseline. PD, Parkinson’s disease; CI, cognitive impairment; pRBD, probable rapid eye movement sleep behavior disorder; and EDS, excessive daytime sleepiness.

Compared to PD patients without fatigue, those with fatigue were more likely to be older (61.49 ± 10.00 vs. 59.88 ± 10.28 years, *p* < 0.001), have a longer disease duration (6.20 ± 4.80 vs. 4.97 ± 4.28 years, *p* < 0.001), and higher baseline LEDD (525.00 ± 280.70 vs. 469.73 ± 275.19 mg, *p* = 0.010). Patients with fatigue had higher total UPDRS, UPDRS part III, bradykinesia, rigidity, tremor, postural instability, total NMSS, total PDQ-39, total SCOPA-AUT, total RBDQ-HK, ESS, and HAMD scores than those without fatigue (all *p* < 0.001, except for tremor score, which showed *p* = 0.140). Patients with fatigue had lower PDSS, total MMSE, and HRS scores than those without fatigue (all *p* < 0.001). Patients with fatigue also showed a higher prevalence of constipation, cognitive problems, hyposmia, pRBD, EDS, depression, wearing-off, and dyskinesia (all *p* < 0.05). In summary, PD patients with fatigue showed more severe motor and non-motor phenotypes (Table 1; Figure 2).

### 3.3. Factors associated with fatigue in PD

Spearman correlation analyses between presence of fatigue and clinical factors are shown in Supplementary Table 3. BMI and the PDSS score (lower score indicate more severe sleep disturbance) were significantly and negatively correlated with the presence of fatigue. Other clinical factors apart from sex were significantly and positively associated with fatigue.

Binary logistic regression analyses were performed to investigate factors associated with fatigue at baseline (Table 2). The variables included in the logistic regression model are mentioned in the Methods section. As shown in Table 2, the univariate logistic regression revealed that age, longer disease duration, LEDD, a greater disease severity (total UPDRS score, Hoehn and Yahr stage), PIGD subtype, total NMSS score, PDSS score, PDQ-39 score, SCOPA-AUT score, presence of FOG, constipation, cognitive impairment, hyposmia, pRBD, EDS, depression, wearing-off and dyskinesia were associated with fatigue (*p* < 0.05). Other clinical variables, including sex and BMI, were not significantly associated with fatigue. We further explored the independent associated factors of fatigue in PD patients using multivariate logistic regression. The total UPDRS score (OR = 1.016, 95% CI: 1.009–1.024, *p* < 0.001), NMSS score (OR = 1.022, 95% CI: 1.015–1.029, *p* < 0.001), PIGD subtype (OR = 1.586, 95% CI: 1.211–2.079, *p* = 0.001), presence of EDS (OR = 1.343, 95% CI: 1.083–1.666, *p* = 0.007), and wearing-off (OR = 1.282, 95% CI: 1.023–1.607, *p* = 0.031) remained significantly associated with fatigue in patients with PD (Table 2).

**Table 2:** Factors Associated with Fatigue in PD.

	Univariate analysis			Multivariate analysis		
Variables (total cohort, *n* = 2,100)	Odds ratio	95% CI	*p* value	Odds ratio	95% CI	*p* value
Sex (women vs. men)	0.983	0.823–1.174	0.849			
BMI	0.985	0.962–1.009	0.219			
Age	1.016	1.007–1.025	**0.001**			
Disease duration	1.062	1.041–1.083	**<0.001**			
LEDD	1.001	1.000–1.001	**<0.001**			
UPDRS total score	1.036	1.031–1.041	**<0.001**	1.016	1.009–1.024	**<0.001**
PIGD subtype (PIGD vs. TD)	2.142	1.690–2.716	**<0.001**	1.586	1.211–2.079	**0.001**
Hoehn and Yahr stages (3–5 vs. 1–2.5)	2.512	2.040–3.095	**<0.001**			
Freezing of gait (presence vs. absence)	1.999	1.646–2.428	**<0.001**			
NMSS total score	1.037	1.033–1.042	**<0.001**	1.022	1.015–1.029	**<0.001**
PDSS total score	0.979	0.975–0.982	**<0.001**			
PDQ-39 total score	1.027	1.023–1.032	**<0.001**			
SCOPA-AUT score	1.088	1.070–1.107	**<0.001**			
Constipation (presence vs. absence)	1.940	1.615–2.331	**<0.001**			
CI (presence vs. absence)	1.990	1.424–2.780	**<0.001**			
Hyposmia (presence vs. absence)	1.589	1.329–1.900	**<0.001**			
pRBD (presence vs. absence)	1.840	1.537–2.203	**<0.001**			
EDS (presence vs. absence)	2.170	1.798–2.619	**<0.001**	1.343	1.083–1.666	**0.007**
Depression (presence vs. absence)	2.988	2.472–3.612	**<0.001**			
Wearing-off (presence vs. absence)	2.013	1.675–2.420	**<0.001**	1.282	1.023–1.607	**0.031**
Dyskinesia (presence vs. absence)	1.709	1.331–2.196	**<0.001**			

*p* value are from logistic regression. Significant *p* values are indicated in bold. PD, Parkinson’s disease; BMI, body mass index; LEDD, levodopa equivalent daily dose; UPDRS, unified Parkinson’s disease rating scale; PIGD, postural instability and gait difficulty; TD, tremor-dominant; NMSS, non-motor symptom scale; PDSS, Parkinson’s disease sleep scale; PDQ-39, The Parkinson’s disease questionnaire (PDQ-39); SCOPA-AUT, scales for outcomes in Parkinson’s disease-autonomic dysfunction; CI, cognitive impairment; pRBD, probable rapid eye movement sleep behavior disorder; EDS, excessive daytime sleepiness.

In order to explore the related factors of fatigue in PD patients at different disease periods, we further performed subgroup analyses according to the disease duration and Hoehn and Yahr stage grouping. The multivariate logistic regression model demonstrated that total UPDRS score (OR = 1.031, 95% CI: 1.013–1.050, *p* < 0.001), NMSS score (OR = 1.026, 95% CI: 1.012–1.040, *p* < 0.001), PIGD subtype (OR = 1.917, 95% CI: 1.185–3.100, *p* = 0.008) and presence of FOG (OR = 0.500, 95% CI: 0.272–0.922, *p* = 0.026) were significantly associated with fatigue in PD patients with disease duration less than or equal to 3 years. More details are shown in Supplementary Table 4.

### 3.4. Predictors of fatigue in PD

The predictors for fatigue in PD are presented in Table 3. In the multivariate model, high total UPDRS score (OR = 1.014, 95% CI: 1.002–1.027, *p* = 0.028) increased the risk of developing fatigue during follow-up.

**Table 3:** Predictors of fatigue in PD.

Variables	Odds ratio	95% CI	*p* value
UPDRS total score	1.014	1.002–1.027	**0.028**

*p* value are from multivariable logistic regression. Significant *p* values are indicated in bold. PD, Parkinson’s disease; UPDRS, Unified Parkinson’s disease Rating Scale.

### 3.5. Disease progression in different fatigue subgroups

Most of the symptoms deteriorated significantly during the follow-up period. Overall, the total UPDRS, UPDRS part III, rigidity, postural instability, NMSS, PDSS, PDQ-39, SCOPA-AUT, MMSE, HRS, RBDQ-HK, and ESS scores worsened significantly from baseline to the 2-year follow-up (*p* < 0.05). The rates of Hoehn and Yahr stages 3–5, freezing of gait, wearing-off, dyskinesia, cognitive impairment, hyposmia, pRBD, and EDS increased significantly (all *p* < 0.05) except constipation and depression. With the persistence of fatigue, most other non-motor symptoms tend to increase in score or prevalence (Supplementary Tables 5 and 6).

Results from the GEE analysis showed significant group progression differences in motor and non-motor symptoms (Table 4 and Supplementary Table 6). In comparison with the never-fatigue group, the persistent-fatigue group showed significantly greater progression in the total UPDRS score (B = 9.477, 95% CI: 7.286–11.668), UPDRS part III score (B = 6.002, 95% CI: 4.507–7.498), and PDQ-39 score (B = 16.882, 95% CI: 13.063–20.701), highlighting the faster disease progression in the persistent-fatigue group (all *p* < 0.001). Similar progression was also observed in several non-motor features, namely, NMSS (B = 21.285, 95% CI: 18.276–24.293), PDSS (B = -10.827, 95% CI: −13.622 to −8.033), SCOPA-AUT (B = 4.147, 95% CI: 3.450–4.844), MMSE (B = -0.515, 95% CI: −0.856 to −0.174), HRS (B = -0.675, 95% CI: −1.286 to −0.065), RBDQ-HK (B = 3.630, 95% CI: 2.134–5.126), ESS (B = 3.203, 95% CI: 2.567–3.840), and HAMD (B = 3.137, 95% CI: 2.534–3.740) scores in the persistent-fatigue group compared to the never-fatigue group. In addition, in comparison with the never-fatigue group, the persistent-fatigue group had a worse prognosis, with more rapid progression in other domains, including freezing of gait (OR = 1.940, 95% CI: 1.541–2.442), wearing-off (OR = 2.255, 95% CI: 1.794–2.835), dyskinesia (OR = 1.971, 95% CI: 1.451–2.676), cognitive impairment (OR = 1.730, 95% CI: 1.227–2.440), pRBD (OR = 1.630, 95% CI: 1.277–2.081), EDS (OR = 2.800, 95% CI: 2.219–3.532), and depression (OR = 3.832, 95% CI: 3.013–4.874). All *p*-values were < 0.001, except that for cognitive impairment (*p* = 0.002).

**Table 4:** Progression of motor and non-motor outcomes in four different fatigue subgroups.

Outcome	Never fatigue (I = 1,142)	Non-persistent fatigue (II = 237)	New-onset fatigue (III = 186)	Persistent fatigue (IV = 535)	*P* value
Motor symptoms					
UPDRS total score	0*	−1.094 (−3.569 to 1.382)	10.616 (7.713 to 13.520)	9.477 (7.286 to 11.668)	*P*_II_ = 0.387, ***P***_III_ **< 0.001,** ***P***_IV_ **< 0.001**
UPDRS Part III score	0*	−1.089 (−2.849 to 0.670)	5.898 (3.870 to 7.926)	6.002 (4.507 to 7.498)	*P*_II_ = 0.225, ***P***_III_ **< 0.001,** ***P***_IV_ **< 0.001**
Bradykinesia score	0*	−0.029 (−0.778 to 0.721)	2.342 (1.448 to 3.236)	2.895 (2.251 to 3.538)	*P*_II_ = 0.940, ***P***_III_ **< 0.001,** ***P***_IV_ **< 0.001**
Rigidity score	0*	−0.256 (−0.784 to 0.272)	1.649 (1.020 to 2.279)	1.586 (1.139 to 2.034)	*P*_II_ = 0.342, ***P***_III_ **< 0.001,** ***P***_IV_ **< 0.001**
Tremor score	0*	−0.617 (−1.028 to −0.207)	0.093 (−0.437 to 0.622)	0.148 (−0.204 to 0.500)	***P***_II_ **= 0.003,** *P*_III_ = 0.732, *P*_IV_=0.410
Postural instability score	0*	0.266 (−0.111 to 0.642)	0.963 (0.536 to 1.390)	0.860 (0.553 to 1.167)	*P*_II_ = 0.167, ***P***_III_ **< 0.001,** ***P***_IV_ **< 0.001**
Freezing of gait^a^	1*	2.308 (1.707 to 3.119)	1.885 (1.346 to 2.641)	1.940 (1.541 to 2.442)	***P***_II_ **< 0.001,** ***P***_III_ **< 0.001,** ***P***_IV_ **< 0.001**
Non-motor symptoms					
NMSS total score	0*	−0.018 (−3.091 to 3.055)	21.940 (17.548 to 26.332)	21.285 (18.276 to 24.293)	***P***_II_= 0.991, ***P***_III_ **< 0.001,** ***P***_IV_ **< 0.001**
PDSS total score	0*	−2.971 (−6.305 to 0.364)	−10.355 (−13.725 to −6.985)	−10.827 (−13.622 to −8.033)	*P*_II_=0.081, ***P***_III_ **< 0.001,** ***P***_IV_ **< 0.001**
PDQ-39 total score	0*	2.592 (−1.148 to 6.332)	16.093 (12.593 to 19.593)	16.882 (13.063 to 20.701)	*P*_II_ = 0.174, ***P***_III_ **< 0.001,** ***P***_IV_ **< 0.001**
SCOPA-AUT score	0*	0.972 (0.195 to 1.750)	3.570 (2.600 to 4.541)	4.147 (3.450 to 4.844)	***P***_II_ **= 0.014,** ***P***_III_ **< 0.001,** ***P***_IV_ **< 0.001**
Constipation^b^	1*	0.996 (0.694 to 1.429)	2.233 (1.566 to 3.185)	2.124 (1.653 to 2.731)	*P*_II_ = 0.982, ***P***_III_ **< 0.001,** ***P***_IV_ **< 0.001**
CI^c^	1*	1.097 (0.635 to 1.894)	1.083 (0.609 to 1.927)	1.730 (1.227 to 2.440)	*P*_II_ = 0.740, *P*_III_ = 0.785, ***P***_IV_**=0.002**
MMSE score	0*	−0.010 (−0.364 to 0.343)	−0.107 (−0.546 to 0.332)	−0.515 (−0.856 to −0.174)	*P*_II_ =0.954, *P*_III_ = 0.632, ***P***_IV_**=0.003**
Hyposmia^d^	1*	1.330 (0.937 to 1.887)	1.553 (1.072 to 2.251)	1.221 (0.955 to 1.562)	*P*_II_ = 0.110, ***P***_III_ **= 0.020,** *P*_IV_ = 0.112
HRS score	0*	−0.601 (−1.398 to 0.195)	−0.806 (−1.699 to 0.087)	−0.675 (−1.286 to −0.065)	*P*_II_ = 0.139, *P*_III_ = 0.077, ***P***_IV_ **= 0.030**
pRBD^e^	1*	1.654 (1.190 to 2.300)	1.907 (1.320 to 2.755)	1.630 (1.277 to 2.081)	***P***_II_**=0.003,** ***P***_III_**=0.001,** ***P***_IV_ **< 0.001**
RBDQ-HK score	0*	1.374 (−0.483 to 3.231)	3.801 (1.559 to 6.043)	3.630 (2.134 to 5.126)	*P*_II_ = 0.147, ***P***_III_**=0.001,** ***P***_IV_ **< 0.001**
EDS^f^	1*	0.895 (0.634 to 1.265)	2.876 (2.029 to 4.077)	2.800 (2.219 to 3.532)	*P*_II_ = 0.531, ***P***_III_ **< 0.001,** ***P***_IV_ **< 0.001**
ESS score	0*	−0.819 (−1.583 to −0.054)	3.514 (2.546 to 4.482)	3.203 (2.567 to 3.840)	*P*_II_ = 0.036, ***P***_III_ **< 0.001,** ***P***_IV_ **< 0.001**
Depression^g^	1*	1.109 (0.775 to 1.587)	3.508 (2.457 to 5.010)	3.832 (3.013 to 4.874)	*P*_II_ = 0.571, ***P***_III_ **< 0.001,** ***P***_IV_ **< 0.001**
HAMD score	0*	0.078 (−0.509 to 0.665)	3.184 (2.391 to 3.977)	3.137 (2.534 to 3.740)	*P*_II_ = 0.794, ***P***_III_ **< 0.001,** ***P***_IV_ **< 0.001**
Motor complications					
Wearing-off^h^	1*	1.288 (0.947 to 1.751)	2.114 (1.503 to 2.973)	2.255 (1.794 to 2.835)	*P*_II_ = 0.107, ***P***_III_ **< 0.001,** ***P***_IV_ **< 0.001**
Dyskinesia^i^	1*	1.335 (0.880 to 2.023)	1.724 (1.080 to 2.752)	1.971 (1.451 to 2.676)	*P*_II_ = 0.174, ***P***_III_**=0.022,** ***P***_IV_ **< 0.001**

Data are B (95%CI) for continuous variables or OR (95%CI) for categorical variables, unless otherwise indicated. In each separate generalized estimating equation, the clinical characteristics at follow-up were defined as dependent variables and the analysis was adjusted for age at baseline, sex, disease duration, and baseline values of the clinical factors. *Reference group. Significant P values are indicated in bold. ^a–i^Evaluated, respectively, by New Freezing of Gait Questionnaire (NFOGQ), Functional Constipation Diagnostic Criteria Rome III (ROME III), MMSE, HRS, RBDQ-HK, ESS, HAMD, 9-item Wearing-off Questionnaire (WOQ-9) and UPDRS part IV-A. PD, Parkinson’s disease; UPDRS, Unified Parkinson’s disease rating scale; NMSS, non-motor symptom scale; PDSS, Parkinson’s disease sleep scale; PDQ-39, The Parkinson’s disease questionnaire (PDQ-39); SCOPA-AUT, scales for outcomes in Parkinson’s disease-autonomic dysfunction; CI, cognitive impairment; MMSE, Mini-mental state examination; HRS, Hyposmia rating scale; pRBD, probable rapid eye movement sleep behavior disorder; RBDQ-HK, rapid eye movement sleep behavior disorder questionnaire-Hong Kong; EDS, excessive daytime sleepiness; ESS, Epworth sleepiness scale; HAMD, Hamilton depression rating scale (HAMD-17).

In comparison with the never-fatigue group, the nonpersistent-fatigue group showed significantly greater progression in the freezing of gait (OR = 2.308, 95% CI: 1.707–3.119), pRBD (OR = 1.654, 95% CI: 1.190–2.300) and total SCOPA-AUT score (B = 0.972, 95% CI: 0.195–1.750). Other factors including total UPDRS, UPDRS part III, NMSS, PDSS, PDQ-39 scores, the rates of constipation, cognitive impairment, hyposmia, EDS, depression, wearing-off, and dyskinesia were not significantly different in the evolution of these symptoms between the never-fatigue and nonpersistent-fatigue group. Additional details are provided in Table 4 and Supplementary Table 6.

## 4. Discussion

This longitudinal cohort study systematically investigated the prevalence, evolution, and clinical correlates of fatigue among PD patients as well as assessed differences in disease progression across PD fatigue subgroups. Overall, approximately one-third of patients included in our study experienced fatigue. We found that PD patients with fatigue were more likely to be older, have a longer disease duration and higher baseline LEDD, and manifested more severe motor and non-motor phenotypes than PD patients without fatigue. We also found that increased disease severity, presence of the PIGD subtype, EDS, and wearing-off were associated with fatigue in patients with PD. To the best of our knowledge, this is the first study to assess longitudinal progression across different fatigue subgroups of PD patients. We observed significant subgroup-level differences in the progression of motor and non-motor symptoms. Our study provides evidence for the longitudinal progression of clinical symptoms in different fatigue subgroups of patients with PD and will facilitate the clinical management of fatigue.

We found that the overall frequency of fatigue in patients with PD was 36.8%, which is consistent with the study of Stocchi et al. (2014) and provides further evidence for fatigue as one of several common non-motor symptoms in patients with PD. The prevalence of fatigue in previous studies (Friedman et al., 2016; Siciliano et al., 2018) has been found to range from 33 to 70%, and the wide range of values may reflect differences in the study designs, especially the use of different fatigue rating scales and definitions of fatigue as well as the populations tested. We also found that the frequency of fatigue increased with the severity of PD, which is consistent with findings from previous studies (Stocchi et al., 2014; Fu et al., 2016; Ou et al., 2021). However, the results of the meta-analysis (Siciliano et al., 2018) showed that the prevalence of fatigue was not moderated by the disease severity.

Our study found that PD patients with fatigue were older, had longer disease duration, and had higher LEDD than those without fatigue. Additionally, PD patients with fatigue showed significantly greater disease severity scores and a higher prevalence of all motor and non-motor symptoms than those without fatigue. These findings indicate that fatigue exacerbates the symptoms and burden of PD patients and are in agreement with previous studies (Stocchi et al., 2014; Fu et al., 2016; Siciliano et al., 2018).

Additionally, we investigated the factors associated with fatigue in order to improve the management of fatigue in patients with PD. In the multivariate logistic regression for the entire cohort, we found that the total UPDRS score, NMSS score and the presence of the PIGD subtype, EDS, and wearing-off were significantly associated with fatigue in patients with PD. Notably, fatigue has been reported to be reduced by dopaminergic drugs, such as levodopa (Lou et al., 2003), suggesting that dopaminergic deficits are involved in the development of fatigue in PD, which is further supported by our finding that more severe motor disabilities were associated with fatigue. However, the non-persistent nature of fatigue observed in many studies (Ongre et al., 2017; Ou et al., 2021; including our study) also suggests that dopaminergic mechanisms only partially contribute to the development of fatigue in PD. In fact, neither LEDD, Hoehn and Yahr stage nor disease duration were associated with fatigue in our study, providing indirect support for the hypothesis that fatigue in PD may result from the disruption of nondopaminergic pathways (Kluger, 2017; Siciliano et al., 2018). This implies that fatigue is independent of dopaminergic treatment in PD and instead an independent non-motor symptom of PD. Although “significant” in UPDRS and NMSS scores associated with fatigue, the odds ratios were close to 1.0 and confidence intervals were narrow, it is likely to be a very small association that may have limited clinical significance. Because UPDRS and NMSS scores are continuous variables, differences in scores between different patients can be large or small. Patients with severe symptoms can roughly be considered as more prone to fatigue than patients with very mild symptoms in clinical practice.

Our study also revealed a relationship between EDS and fatigue, which is consistent with the results of previous studies (Stocchi et al., 2014; Siciliano et al., 2018). Nevertheless, there are a few inconsistent findings in our study regarding factors associated with fatigue that should be noted. For example, some studies have shown that poor quality of life, sleep disorders, and depression (Ongre et al., 2021; Ou et al., 2021; Béreau et al., 2022) are related to fatigue, whereas we did not find an association between fatigue and the patients’ PDQ-39 score, PDSS score, the presence of pRBD, or depression as a whole. In order to determine whether the results of related factors are different due to different disease stages, we performed subgroup analyses of correlated factors grouped by disease duration and Hoehn and Yahr stages. We found that the total UPDRS score, NMSS score, PIGD subtype, and the absence of FOG were significantly associated with fatigue in PD patients with disease duration less than or equal to 3 years. The PDSS score was found to be associated with fatigue in patients with a disease course longer than 10 years; however, the OR value was close to 1 with little effect. No relationship was found between fatigue and PDQ-39, pRBD, or depression in each subgroup analysis, which may be ascribed to different inclusion criteria and confounding factors considered in previous studies (Ongre et al., 2021; Ou et al., 2021; Béreau et al., 2022). Our findings also differed from those of previous studies in that we found that the presence of the PIGD subtype and wearing-off were associated with fatigue. The link between fatigue and wearing-off further implicates that dopaminergic factors—either *via* direct dopaminergic neurotransmission or *via* indirect mechanisms—are key for the development of fatigue (Franke and Storch, 2017). Unfortunately, to date, there is no effective treatment for fatigue in patients with PD (Elbers et al., 2016), and rasagiline was only proposed as “possibly useful” (Seppi et al., 2019), deep brain stimulation may help improve fatigue in patients with PD (Lazcano-Ocampo et al., 2021). Therefore, PD patients with fatigue may benefit from the management of motor symptoms, wearing-off, and EDS because they are all associated with fatigue in these patients.

Our study also demonstrated that nearly all PD motor and non-motor symptoms significantly deteriorated over time, and that the occurrence of fatigue in PD was associated with worse baseline findings and faster progression rates, which are consistent with findings from previous studies. For example, data from the Oxford Parkinson’s Disease Centre Discovery cohort demonstrated that almost all PD motor and non-motor symptoms measured in the study significantly increased over time (Liu et al., 2021). Other studies have also confirmed the longitudinal progression of both motor and non-motor symptoms, including autonomic dysfunction, EDS, and cognitive impairment (Amara et al., 2017; Simuni et al., 2018; Stanković et al., 2019; Myers et al., 2022). It should be noted that a subset of symptoms have shown stability, relief, and improvement in longitudinal studies (Cilia et al., 2020; Simuni et al., 2022). However, despite these inconsistencies, most studies have reported an overall deterioration of motor and non-motor symptoms, consistent with our data. In the subgroup analysis of our study, the persistent-fatigue group showed significantly greater progression in most symptoms, in comparison with the never-fatigue group. In comparison with the never-fatigue group, the nonpersistent-fatigue group showed more rapid progression in the freezing of gait, pRBD and total SCOPA-AUT score. However, other clinical features (including motor symptoms, wearing-off, dyskinesia, EDS, depression, quality of life and cognitive symptoms) were not significantly different in the evolution of these symptoms between these two groups. This reflected indirectly that the prognosis of patients with non-persistent fatigue was better than that of patients with persistent fatigue in the overall cohort, which further proves that fatigue can aggravate disease progression. Moreover, this suggests that the improvement of fatigue may alleviate motor and other non-motor symptoms to some extent. The results also provide indirect support for the hypothesis that progression during early PD in the persistent fatigue group may be faster than that in the non-persistent fatigue group.

The relationship between treatment and relief or aggravation of fatigue has not been thoroughly studied in the past. The factors influencing fatigue are quite numerous, and may include (in addition to the drugs regime) the amount of physical exercise and physical labor, emotional aspects, and diet, among others (Fu et al., 2016; Ongre et al., 2017; Lin et al., 2021; Ongre et al., 2021). A previous study analyzed factors associated with fatigue improvement, and found that patients with fatigue at baseline received higher doses of dopaminergic medication during follow-up than those without fatigue. Moreover, the study showed that the improvement of fatigue could not necessarily be attributed to changes in disease severity or other non-motor symptoms (such as depressive symptoms, sleep disorder, apathy, or cognitive impairment), and suggested an improved efficacy of dopamine agonists over levodopa in PD patients with fatigue (Ongre et al., 2017). However, the authors of this study did not take into account some additional confounding factors. Rigorous prospective cohort studies, with very strict design, precise group allocation, and with more confounding factors being considered, are needed in the future to further explore the relationship between the drug regime or other alleviating/aggravation factors, and the evolution of fatigue. Proposed pathophysiologic mechanisms of fatigue currently include increased circulating proinflammatory cytokines, dysfunction in nigrostriatal and extrastriatal dopaminergic pathways, involvement of nondopaminergic (particularly serotonergic) pathways, executive/prefrontal pathology, and involvement of the autonomic nervous system (Kluger, 2017; Lazcano-Ocampo et al., 2020). Further longitudinal studies are needed to corroborate our results and to investigate the specific neurobiological pathways related to fatigue in PD. To the best of our knowledge, no other studies have evaluated the longitudinal progression in PD patients with and without fatigue. The above findings suggest that screening for fatigue symptoms at baseline could improve the estimation of the subsequent disease trajectory, enabling better individualized treatment, information, and support to be offered to patients and their caregivers.

This study had some limitations. First, although the 2-year follow-up period was relatively short, we plan to address this by continuing to follow up our cohort of patients. Second, our study involved heterogeneity in clinical data collection and the data quality obtained from different investigators. Nevertheless, all researchers involved in the study received standardized and unified training to ensure consistency and quality of the data as much as possible. Third, PFS-16 was developed to assess a single construct reflecting the physical aspects of fatigue in patients with PD and to measure both the presence of fatigue and its severity. But this scale may not adequately reflect clinically significant non-physical aspects of fatigue and does not define fatigue. However, PFS-16 is a brief and easily completed scale developed specifically for PD, and meets the criteria for the designation of “recommended” as defined by the Movement Disorder Society for screening fatigue (Friedman et al., 2010). Fourth, the study was lacking in biomarkers for the characterization of fatigue. Future studies combining clinical information and additional variables (i.e., genetics, neuroimaging markers, and other biomarkers) are warranted.

## 5. Conclusion

Increased disease severity, presence of the PIGD subtype, EDS, and wearing-off were associated with fatigue in patients with PD. Significant subgroup-level differences were observed in the progression of motor and non-motor symptoms across different fatigue subgroups of PD patients.

## Data availability statement

The raw data supporting the conclusions of this article will be made available by the authors, without undue reservation.

## Ethics statement

The studies involving human participants were reviewed and approved by Ethics Committee of Xiangya Hospital of Central South University. The patients/participants provided their written informed consent to participate in this study.

## Author contributions

XZ: research project conception, organization, execution, statistical analysis design and execution of the study, patients’ enrollment and follow up, and writing of the first manuscript draft. YX, TS, YZ, and HP: research project execution and patients’ enrollment and follow up. QX, YC, QS, XY, JG, and BT: research project organization, execution, and manuscript review and critique. XW: statistical analysis design, execution, review and critique of the study and manuscript review and critique. LL and ZL: research project conception, organization, execution, and manuscript review and critique. All authors contributed to the article and approved the submitted version.

## Funding

This study was supported by the Hunan Innovative Province Construction Project (Grant Nos. 2019SK2335 and 2021SK1010), Natural Science Foundation of Hunan Province (Grant No. 2022JJ40843), National Key Research and Development Program of China (Grant Nos. 2021YFC2501204 and 2016YFC306000), National Natural Science Foundation of China (Grant No. 82001359), and Scientific Research Project of Hunan Provincial Health Commission (Grant No. 202203074637).

## Conflict of interest

The authors declare that the research was conducted in the absence of any commercial or financial relationships that could be construed as a potential conflict of interest.

## Publisher’s note

All claims expressed in this article are solely those of the authors and do not necessarily represent those of their affiliated organizations, or those of the publisher, the editors and the reviewers. Any product that may be evaluated in this article, or claim that may be made by its manufacturer, is not guaranteed or endorsed by the publisher.

## Footnotes

^1^ http://www.pd-mdcnc.com
