# Supplementary material for: Characteristics of fatigue in Parkinson’s disease: A longitudinal cohort study
Source: Front Aging Neurosci. 2023 Mar 10;15:1133705. doi: 10.3389/fnagi.2023.1133705 (PMC10036570; doi:10.3389/fnagi.2023.1133705)
Supplement: Supplementary file 1 [file Table_1.DOCX]

Supplementary Material

**Supplementary Table 1. Prevalence of fatigue in PD at different disease duration and Hoehn and Yahr stages**

| **Grouping** | **Prevalence of fatigue, %** | ***P* value** |
| --- | --- | --- |
| **Subgroup at disease duration** |  | <**0.001** |
| Disease duration**≤**3 years (n=728) | 192(26.4%) |  |
| 3＜Disease duration＜10 years (n=1096) | 452(41.2%) |  |
| Disease duration≥10 years (n=276) | 128(46.4%) |  |
| **Subgroup at disease stage** |  | <**0.001** |
| Hoehn and Yahr stages of 1-2.5 (n=1625) | 103(31.8%) |  |
| Hoehn and Yahr stages of 3-5 (n=475) | 103(53.9%) |  |
| **Total (n=2100)** | 772(36.8%) |  |

**Supplementary Table 2. Demographic and clinical characteristics of patients with PD at baseline at different disease duration**

| **Characteristics** | **Overall (n=2100)** | **DD≤3 (n=728)** | **3＜DD＜10 (n=1096)** | **DD＞10 (n=276)** | ***P* value** |
| --- | --- | --- | --- | --- | --- |
| Age at baseline | 60.47±10.21 | 59.30±10.43 | 60.76±10.03 | 62.45±9.99 | **＜0.001** |
| Age at onset | 55.06±10.81 | 57.65±10.39 | 55.06±10.12 | 48.22±11.62 | **＜0.001** |
| Gender ratio (male) | 1047(49.9%) | 370(50.8%) | 546(49.8%) | 131(47.5%) | 0.636 |
| BMI | 22.78±5.22 | 23.24±7.47 | 22.69±3.39 | 21.92±3.63 | **＜0.001** |
| PD duration | 5.42±4.52 | 1.65±0.75 | 5.71±1.81 | 14.23±5.02 | **＜0.001** |
| LEDD | 490.05±278.44 | 393.70±233.73 | 511.43±275.53 | 659.31±299.79 | **＜0.001** |
| **Motor symptoms** |  |  |  |  |  |
| UPDRS total score | 39.86±20.40 | 30.15±14.26 | 42.11±19.14 | 56.52±25.17 | **＜0.001** |
| UPDRS Part III score | 25.15±13.97 | 19.47±10.01 | 26.48±13.45 | 34.87±17.79 | **＜0.001** |
| Bradykinesia score | 9.29±6.00 | 7.34±4.58 | 9.67±5.94 | 12.93±7.40 | **＜0.001** |
| Rigidity score | 5.19±4.00 | 3.95±3.11 | 5.66±4.04 | 6.55±4.97 | **＜0.001** |
| Tremor score | 3.33±3.30 | 2.64±2.27 | 3.44±3.37 | 4.72±4.60 | **＜0.001** |
| Postural instability score | 3.73±2.76 | 2.65±1.96 | 3.89±2.57 | 5.95±3.65 | **＜0.001** |
| Motor subtype |  |  |  |  | **＜0.001** |
| Tremor-dominant | 460(21.9%) | 199(27.3%) | 220(20.1%) | 41(14.9%) |  |
| Intermediate type | 291(13.9%) | 105(14.5%) | 154(14.0%) | 32(11.5%) |  |
| PIGD-dominant | 1349(64.2%) | 424(58.2%) | 722(65.9%) | 203(73.6%) |  |
| Hoehn and Yahr stages |  |  |  |  | **＜0.001** |
| stages of 1-2.5 | 1625(77.4%) | 667(91.6%) | 829(75.6%) | 129(46.7%) |  |
| stages of 3-5 | 475(22.6%) | 61(8.4%) | 267(24.4%) | 147(53.3%) |  |
| Freezing of gait^a^ | 588(28.0%) | 95(13.0%) | 350(31.9%) | 143(51.8%) | **＜0.001** |
| **Non-motor symptoms** |  |  |  |  |  |
| NMSS total score | 33.96±25.55 | 26.53±21.91 | 35.84±24.59 | 46.08±31.59 | **＜0.001** |
| PDSS total score | 119.28±28.60 | 124.68±22.93 | 118.75±30.83 | 107.10±29.00 | **＜0.001** |
| PDQ-39 total score | 26.09±31.46 | 16.63±17.49 | 27.77±34.37 | 44.42±38.15 | **＜0.001** |
| SCOPA-AUT score | 8.07±5.42 | 6.80±5.10 | 8.43±5.23 | 9.98±6.16 | **＜0.001** |
| Constipation^b^ | 760(36.2%) | 182(25.0%) | 419(38.2%) | 159(57.6%) | **＜0.001** |
| CI^c^ | 149(7.1%) | 26(3.6%) | 94(8.6%) | 29(10.5%) | **＜0.001** |
| MMSE score | 26.73±3.37 | 27.13±2.98 | 26.65±3.46 | 26.04±3.80 | **＜0.001** |
| Hyposmia^d^ | 899(42.8%) | 270(37.1%) | 490(44.7%) | 139(50.4%) | **＜0.001** |
| HRS score | 19.58±6.22 | 20.21±5.95 | 19.45±6.19 | 18.39±6.81 | **＜0.001** |
| pRBD^e^ | 882(42.0%) | 211(29.0%) | 513(46.8%) | 158(57.2%) | **＜0.001** |
| RBDQ-HK score | 16.32±16.43 | 11.91±13.94 | 17.48±16.51 | 23.36±18.84 | **＜0.001** |
| EDS^f^ | 677(32.2%) | 198(27.2%) | 367(33.5%) | 112(40.6%) | **＜0.001** |
| ESS score | 7.37±6.16 | 6.35±5.77 | 7.66±6.21 | 8.88±6.53 | **＜0.001** |
| Depression^g^ | 685(32.6%) | 175(24.0%) | 383(34.9%) | 127(46.0%) | **＜0.001** |
| HAMD score | 5.31±5.19 | 4.31±4.76 | 5.56±5.11 | 6.94±5.99 | **＜0.001** |
| Fatigue^h^ | 772(36.8%) | 192(26.4%) | 452(41.2%) | 128(46.4%) | **＜0.001** |
| PFS-16 total score | 44.01±18.59 | 39.58±17.49 | 45.85±18.60 | 48.37±19.20 | **＜0.001** |
| **Motor complications** |  | | | | |
| Wearing-off^i^ | 757(36.0%) | 109(15.0%) | 468(42.7%) | 180(65.2%) | **＜0.001** |
| Dyskinesia^j^ | 288(13.7%) | 16(2.2%) | 165(15.1%) | 107(38.8%) | **＜0.001** |

Data are mean ± SD or n (%), unless otherwise indicated. P value are from the chi-squared test, Student’s t-test or nonparametric Mann–Whitney test. Significant P values are indicated in bold. a-j Evaluated respectively by New Freezing of Gait Questionnaire (NFOGQ), Functional Constipation Diagnostic Criteria Rome Ⅲ (ROME III), MMSE, HRS, RBDQ-HK, ESS, HAMD, PFS-16, 9-item Wearing-off Questionnaire (WOQ-9) and UPDRS part IV-A. PD, Parkinson’s disease; DD, disease duration; BMI, Body Mass Index; LEDD, levodopa equivalent daily dose; UPDRS, Unified Parkinson’s disease Rating Scale; PIGD, postural instability and gait difficulty; NMSS, Non-Motor Symptom Scale; PDSS, Parkinson’s Disease Sleep Scale; PDQ-39, The Parkinson's Disease Questionnaire (PDQ-39); SCOPA-AUT, Scales for outcomes in Parkinson’s disease-Autonomic Dysfunction; CI, Cognitive Impairment; MMSE, Mini-Mental State Examination; HRS, Hyposmia Rating Scale; pRBD, probable rapid eye movement sleep behavior disorder; RBDQ-HK, Rapid Eye Movement Sleep Behavior Disorder Questionnaire-Hong Kong; EDS, Excessive daytime sleepiness; ESS, Epworth Sleepiness Scale; HAMD, Hamilton Depression Rating Scale (HAMD-17); PFS-16, the 16-item Parkinson’s Fatigue Scale (PFS-16) questionnaire.

**Supplementary Table 3. Correlations between fatigue and clinical characteristics of the patients with PD**

| **Variables** | **Spearman’s correlation coefficient** | ***P* value** |
| --- | --- | --- |
| Sex | -0.004 | 0.849 |
| BMI | -0.076 | <**0.001** |
| Age | 0.077 | <**0.001** |
| Disease duration | 0.167 | <**0.001** |
| LEDD | 0.103 | <**0.001** |
| UPDRS total score | 0.343 | <**0.001** |
| PIGD subtype | 0.143 | <**0.001** |
| Hoehn and Yahr stages | 0.192 | <**0.001** |
| Freezing of gait | 0.154 | <**0.001** |
| NMSS total score | 0.394 | <**0.001** |
| PDSS total score | -0.286 | <**0.001** |
| PDQ-39 total score | 0.358 | <**0.001** |
| SCOPA-AUT score | 0.214 | <**0.001** |
| Constipation | 0.155 | <**0.001** |
| CI | 0.089 | <**0.001** |
| Hyposmia | 0.111 | <**0.001** |
| pRBD | 0.146 | <**0.001** |
| EDS | 0.178 | <**0.001** |
| Depression | 0.251 | <**0.001** |
| Wearing-off | 0.164 | <**0.001** |
| Dyskinesia | 0.092 | <**0.001** |

BMI, Body Mass Index; LEDD, levodopa equivalent daily dose; UPDRS, Unified Parkinson’s disease Rating Scale; PIGD, postural instability and gait difficulty; NMSS, Non-Motor Symptom Scale; PDSS, Parkinson’s Disease Sleep Scale; PDQ-39, The Parkinson's Disease Questionnaire (PDQ-39); SCOPA-AUT, Scales for outcomes in Parkinson’s disease-Autonomic Dysfunction; CI, Cognitive Impairment; pRBD, probable rapid eye movement sleep behavior disorder; EDS, Excessive daytime sleepiness.

**Supplementary Table 4. Factors associated with fatigue in PD at different disease duration and Hoehn and Yahr stages**

|  | **Univariate analysis** | | |  | **Multivariate analysis** | | |
| --- | --- | --- | --- | --- | --- | --- | --- |
| **Variables** | **Odds ratio** | **95% CI** | ***P* value** |  | **Odds ratio** | **95% CI** | ***P* value** |
| **Disease duration ≤3 years (n=728)** |  |  |  | |  |  |  |
| Sex (women vs. men) | 0.988 | 0.711-1.374 | 0.944 | |  |  |  |
| BMI | 1.010 | 0.989-1.032 | 0.347 | |  |  |  |
| Age | 1.007 | 0.991-1.023 | 0.371 | |  |  |  |
| Disease duration | 1.285 | 1.030-1.602 | **0.026** | |  |  |  |
| LEDD | 1.001 | 1.000-1.001 | 0.156 | |  |  |  |
| UPDRS total score | 1.046 | 1.033-1.059 | <**0.001** | | 1.031 | 1.013-1.050 | **0.001** |
| PIGD subtype (PIGD vs. TD) | 2.224 | 1.452-3.408 | <**0.001** | | 1.917 | 1.185-3.100 | **0.008** |
| Hoehn and Yahr stages (3-5 vs. 1-2.5) | 1.523 | 0.873-2.655 | 0.138 | |  |  |  |
| Freezing of gait (presence vs. absence) | 1.342 | 0.840-2.143 | 0.218 | | 0.500 | 0.272-0.922 | **0.026** |
| NMSS total score | 1.041 | 1.031-1.050 | <**0.001** | | 1.026 | 1.012-1.040 | <**0.001** |
| PDSS total score | 0.981 | 0.974-0.988 | <**0.001** | |  |  |  |
| PDQ-39 total score | 1.035 | 1.025-1.045 | <**0.001** | |  |  |  |
| SCOPA-AUT score | 1.074 | 1.041-1.109 | <**0.001** | |  |  |  |
| Constipation (presence vs. absence) | 1.711 | 1.190-2.462 | **0.004** | |  |  |  |
| CI (presence vs. absence) | 1.251 | 0.535-2.926 | 0.605 | |  |  |  |
| Hyposmia (presence vs. absence) | 1.421 | 1.015-1.989 | **0.041** | |  |  |  |
| pRBD (presence vs. absence) | 1.826 | 1.288-2.590 | **0.001** | |  |  |  |
| EDS (presence vs. absence) | 2.223 | 1.562-3.164 | <**0.001** | |  |  |  |
| Depression (presence vs. absence) | 2.993 | 2.083-4.299 | <**0.001** | |  |  |  |
| Wearing-off (presence vs. absence) | 1.256 | 0.804-1.963 | 0.317 | |  |  |  |
| Dyskinesia (presence vs. absence) | 2.216 | 0.814-6.034 | 0.120 | |  |  |  |
| **3＜Disease duration＜10 years (n=1096)** |  |  |  | |  |  |  |
| Sex (women vs. men) | 1.018 | 0.800-1.295 | 0.885 | |  |  |  |
| BMI | 0.963 | 0.930-0.999 | **0.041** | |  |  |  |
| Age | 1.010 | 0.998-1.022 | 0.103 | |  |  |  |
| Disease duration | 1.050 | 0.983-1.122 | 0.148 | |  |  |  |
| LEDD | 1.000 | 1.000-1.001 | **0.048** | |  |  |  |
| UPDRS total score | 1.035 | 1.027-1.042 | <**0.001** | | 1.016 | 1.006-1.027 | **0.002** |
| PIGD subtype (PIGD vs. TD) | 1.786 | 1.299-2.456 | <**0.001** | |  |  |  |
| Hoehn and Yahr stages (3-5 vs. 1-2.5) | 2.283 | 1.725-3.022 | <**0.001** | |  |  |  |
| Freezing of gait (presence vs. absence) | 1.722 | 1.332-2.227 | <**0.001** | |  |  |  |
| NMSS total score | 1.035 | 1.028-1.041 | <**0.001** | | 1.020 | 1.011-1.029 | <**0.001** |
| PDSS total score | 0.981 | 0.976-0.987 | <**0.001** | |  |  |  |
| PDQ-39 total score | 1.022 | 1.016-1.028 | <**0.001** | |  |  |  |
| SCOPA-AUT score | 1.079 | 1.053-1.106 | <**0.001** | |  |  |  |
| Constipation (presence vs. absence) | 1.722 | 1.344-2.205 | <**0.001** | |  |  |  |
| CI (presence vs. absence) | 2.049 | 1.335-3.143 | **0.001** | | 1.707 | 1.051-2.774 | **0.031** |
| Hyposmia (presence vs. absence) | 1.602 | 1.257-2.042 | <**0.001** | |  |  |  |
| pRBD (presence vs. absence) | 1.538 | 1.208-1.960 | <**0.001** | |  |  |  |
| EDS (presence vs. absence) | 2.154 | 1.668-2.781 | <**0.001** | | 1.430 | 1.067-1.916 | **0.017** |
| Depression (presence vs. absence) | 2.798 | 2.166-3.615 | <**0.001** | |  |  |  |
| Wearing-off (presence vs. absence) | 1.823 | 1.428-2.328 | <**0.001** | | 1.424 | 1.070-1.895 | **0.015** |
| Dyskinesia (presence vs. absence) | 1.335 | 0.958-1.862 | 0.088 | |  |  |  |
| **Disease duration≥10 years (n=276)** |  |  |  | |  |  |  |
| Sex (women vs. men) | 0.780 | 0.485-1.254 | 0.305 | | 0.492 | 0.270-0.899 | **0.021** |
| BMI | 0.931 | 0.870-0.996 | **0.039** | | 0.891 | 0.820-0.967 | **0.006** |
| Age | 1.039 | 1.014-1.066 | **0.003** | |  |  |  |
| Disease duration | 1.003 | 0.975-1.051 | 0.902 | |  |  |  |
| LEDD | 1.000 | 0.999-1.001 | 0.609 | |  |  |  |
| UPDRS total score | 1.025 | 1.014-1.036 | <**0.001** | |  |  |  |
| PIGD subtype (PIGD vs. TD) | 2.648 | 1.259-5.570 | **0.010** | |  |  |  |
| Hoehn and Yahr stages (3-5 vs. 1-2.5) | 2.576 | 1.579-4.201 | <**0.001** | |  |  |  |
| Freezing of gait (presence vs. absence) | 2.249 | 1.386-3.648 | **0.001** | |  |  |  |
| NMSS total score | 1.033 | 1.022-1.044 | <**0.001** | | 1.017 | 1.001-1.034 | **0.040** |
| PDSS total score | 0.975 | 0.966-0.985 | <**0.001** | | 0.982 | 0.970-0.994 | **0.003** |
| PDQ-39 total score | 1.026 | 1.016-1.035 | <**0.001** | |  |  |  |
| SCOPA-AUT score | 1.093 | 1.047-1.141 | <**0.001** | |  |  |  |
| Constipation (presence vs. absence) | 1.977 | 1.213-3.221 | **0.006** | |  |  |  |
| CI (presence vs. absence) | 1.484 | 0.684-3.215 | 0.318 | |  |  |  |
| Hyposmia (presence vs. absence) | 1.382 | 0.860-2.221 | 0.182 | |  |  |  |
| pRBD (presence vs. absence) | 1.795 | 1.105-2.917 | **0.018** | |  |  |  |
| EDS (presence vs. absence) | 1.629 | 1.004-2.643 | **0.048** | |  |  |  |
| Depression (presence vs. absence) | 2.456 | 1.511-3.993 | <**0.001** | |  |  |  |
| Wearing-off (presence vs. absence) | 1.863 | 1.121-3.097 | **0.016** | |  |  |  |
| Dyskinesia (presence vs. absence) | 1.230 | 0.757-1.999 | 0.403 | |  |  |  |
| **Hoehn and Yahr stages of 1-2.5 (n=1625)** |  |  |  | |  |  |  |
| Sex (women vs. men) | 1.001 | 0.812-1.234 | 0.992 | |  |  |  |
| BMI | 0.988 | 0.962-1.015 | 0.383 | |  |  |  |
| Age | 1.011 | 1.001-1.022 | **0.035** | | **0.986** | **0.974-0.999** | **0.036** |
| Disease duration | 1.045 | 1.016-1.074 | **0.002** | |  |  |  |
| LEDD | 1.001 | 1.000-1.001 | **0.004** | |  |  |  |
| UPDRS total score | 1.047 | 1.039-1.055 | <**0.001** | | 1.030 | 1.020-1.040 | <**0.001** |
| PIGD subtype (PIGD vs. TD) | 1.902 | 1.466-2.467 | <**0.001** | | 1.611 | 1.199-2.163 | **0.002** |
| Hoehn and Yahr stages (3-5 vs. 1-2.5) | NA | NA | NA | |  |  |  |
| Freezing of gait (presence vs. absence) | 1.502 | 1.167-1.934 | **0.002** | | 0.628 | 0.456-0.863 | **0.004** |
| NMSS total score | 1.042 | 1.036-1.048 | <**0.001** | | 1.026 | 1.018-1.035 | <**0.001** |
| PDSS total score | 0.982 | 0.977-0.986 | <**0.001** | |  |  |  |
| PDQ-39 total score | 1.031 | 1.024-1.037 | <**0.001** | |  |  |  |
| SCOPA-AUT score | 1.081 | 1.058-1.105 | <**0.001** | |  |  |  |
| Constipation (presence vs. absence) | 1.975 | 1.587-2.457 | <**0.001** | |  |  |  |
| CI (presence vs. absence) | 1.494 | 0.965-2.313 | 0.072 | |  |  |  |
| Hyposmia (presence vs. absence) | 1.530 | 1.239-1.889 | <**0.001** | |  |  |  |
| pRBD (presence vs. absence) | 1.719 | 1.391-2.126 | <**0.001** | |  |  |  |
| EDS (presence vs. absence) | 1.986 | 1.589-2.483 | <**0.001** | | 1.317 | 1.020-1.700 | **0.035** |
| Depression (presence vs. absence) | 2.934 | 2.330-3.694 | <**0.001** | |  |  |  |
| Wearing-off (presence vs. absence) | 1.714 | 1.372-2.141 | <**0.001** | |  |  |  |
| Dyskinesia (presence vs. absence) | 1.777 | 1.296-2.438 | <**0.001** | |  |  |  |
| **Hoehn and Yahr stages of 3-5 (n=475)** |  |  |  | |  |  |  |
| Sex (women vs. men) | 0.749 | 0.520-1.080 | 0.121 | |  |  |  |
| BMI | 1.003 | 0.952-1.055 | 0.920 | |  |  |  |
| Age | 1.008 | 0.990-1.026 | 0.406 | |  |  |  |
| Disease duration | 1.022 | 0.990-1.056 | 0.180 | |  |  |  |
| LEDD | 1.000 | 1.000-1.001 | 0.326 | |  |  |  |
| UPDRS total score | 1.018 | 1.009-1.026 | <**0.001** | |  |  |  |
| PIGD subtype (PIGD vs. TD) | 1.420 | 0.709-2.844 | 0.323 | |  |  |  |
| Hoehn and Yahr stages (3-5 vs. 1-2.5) | NA | NA | NA | |  |  |  |
| Freezing of gait (presence vs. absence) | 1.813 | 1.257-2.614 | **0.001** | |  |  |  |
| NMSS total score | 1.024 | 1.016-1.031 | <**0.001** | | 1.015 | 1.004-1.025 | **0.007** |
| PDSS total score | 0.980 | 0.972-0.987 | <**0.001** | |  |  |  |
| PDQ-39 total score | 1.015 | 1.008-1.022 | <**0.001** | |  |  |  |
| SCOPA-AUT score | 1.066 | 1.033-1.099 | <**0.001** | |  |  |  |
| Constipation (presence vs. absence) | 1.307 | 0.910-1.877 | 0.147 | |  |  |  |
| CI (presence vs. absence) | 2.192 | 1.221-3.933 | **0.009** | | 1.987 | 1.027-3.845 | **0.042** |
| Hyposmia (presence vs. absence) | 1.630 | 1.132-2.347 | **0.009** | |  |  |  |
| pRBD (presence vs. absence) | 1.789 | 1.242-2.576 | **0.002** | |  |  |  |
| EDS (presence vs. absence) | 2.151 | 1.479-3.127 | <**0.001** | |  |  |  |
| Depression (presence vs. absence) | 1.915 | 1.327-2.763 | **0.001** | |  |  |  |
| Wearing-off (presence vs. absence) | 1.804 | 1.250-2.604 | **0.002** | |  |  |  |
| Dyskinesia (presence vs. absence) | 1.062 | 0.691-1.632 | 0.784 | |  |  |  |
| **Total cohort (n=2100)** |  |  |  | |  |  |  |
| Sex (women vs. men) | 0.983 | 0.823-1.174 | 0.849 | |  |  |  |
| BMI | 0.985 | 0.962-1.009 | 0.219 | |  |  |  |
| Age | 1.016 | 1.007-1.025 | **0.001** | |  |  |  |
| Disease duration | 1.062 | 1.041-1.083 | <**0.001** | |  |  |  |
| LEDD | 1.001 | 1.000-1.001 | <**0.001** | |  |  |  |
| UPDRS total score | 1.036 | 1.031-1.041 | <**0.001** | | 1.016 | 1.009-1.024 | <**0.001** |
| PIGD subtype (PIGD vs. TD) | 2.142 | 1.690-2.716 | <**0.001** | | 1.586 | 1.211-2.079 | **0.001** |
| Hoehn and Yahr stages (3-5 vs. 1-2.5) | 2.512 | 2.040-3.095 | <**0.001** | |  |  |  |
| Freezing of gait (presence vs. absence) | 1.999 | 1.646-2.428 | <**0.001** | |  |  |  |
| NMSS total score | 1.037 | 1.033-1.042 | <**0.001** | | 1.022 | 1.015-1.029 | <**0.001** |
| PDSS total score | 0.979 | 0.975-0.982 | <**0.001** | |  |  |  |
| PDQ-39 total score | 1.027 | 1.023-1.032 | <**0.001** | |  |  |  |
| SCOPA-AUT score | 1.088 | 1.070-1.107 | <**0.001** | |  |  |  |
| Constipation (presence vs. absence) | 1.940 | 1.615-2.331 | <**0.001** | |  |  |  |
| CI (presence vs. absence) | 1.990 | 1.424-2.780 | <**0.001** | |  |  |  |
| Hyposmia (presence vs. absence) | 1.589 | 1.329-1.900 | <**0.001** | |  |  |  |
| pRBD (presence vs. absence) | 1.840 | 1.537-2.203 | <**0.001** | |  |  |  |
| EDS (presence vs. absence) | 2.170 | 1.798-2.619 | <**0.001** | | 1.343 | 1.083-1.666 | **0.007** |
| Depression (presence vs. absence) | 2.988 | 2.472-3.612 | <**0.001** | |  |  |  |
| Wearing-off (presence vs. absence) | 2.013 | 1.675-2.420 | <**0.001** | | 1.282 | 1.023-1.607 | **0.031** |
| Dyskinesia (presence vs. absence) | 1.709 | 1.331-2.196 | <**0.001** | |  |  |  |

*P* value are from logistic regression. Significant *P* values are indicated in bold. PD, Parkinson’s disease; BMI, Body Mass Index; LEDD, levodopa equivalent daily dose; UPDRS, Unified Parkinson’s disease Rating Scale; PIGD, postural instability and gait difficulty; TD, Tremor-dominant; NMSS, Non-Motor Symptom Scale; PDSS, Parkinson’s Disease Sleep Scale; PDQ-39, The Parkinson's Disease Questionnaire (PDQ-39); SCOPA-AUT, Scales for outcomes in Parkinson’s disease-Autonomic Dysfunction; CI, Cognitive Impairment; pRBD, probable rapid eye movement sleep behavior disorder; EDS, Excessive daytime sleepiness.

**Supplementary Table 5. Clinical features of patients with PD at baseline and follow-up**

| **Characteristics** | **Baseline (n=2100)** | **Follow-up (n=2100)** | ***P* value** |
| --- | --- | --- | --- |
| **Motor symptoms** |  |  |  |
| UPDRS total score | 39.86±20.40 | 43.32±23.97 | **＜0.001** |
| UPDRS Part III score | 25.15±13.97 | 26.53±15.93 | **＜0.001** |
| Bradykinesia score | 9.29±6.00 | 9.62±6.63 | 0.090 |
| Rigidity score | 5.19±4.00 | 5.54±4.35 | **0.001** |
| Tremor score | 3.33±3.31 | 3.36±3.59 | 0.897 |
| Postural instability score | 3.73±2.76 | 4.18±3.33 | **＜0.001** |
| Hoehn and Yahr stages |  |  | **＜0.001** |
| stages of 1-2.5 | 1625(77.4%) | 1491(71.0%) |  |
| stages of 3-5 | 475(22.6%) | 609(29.0%) |  |
| Freezing of gait^a^ | 588(28.0%) | 838(39.9%) | **＜0.001** |
| **Nonmotor symptoms** |  |  |  |
| NMSS total score | 33.96±25.55 | 37.26±30.74 | **＜0.001** |
| PDSS total score | 119.28±28.60 | 117.64±24.86 | **0.009** |
| PDQ-39 total score | 26.09±31.46 | 28.42±25.92 | **＜0.001** |
| SCOPA-AUT score | 8.07±5.42 | 9.11±6.97 | **＜0.001** |
| Constipation^b^ | 760(36.2%) | 625(29.8%) | **＜0.001** |
| CI^c^ | 149(7.1%) | 220(10.5%) | **＜0.001** |
| MMSE score | 26.73±3.37 | 26.53±3.86 | **0.032** |
| Hyposmia^d^ | 899(42.8%) | 1055(50.2%) | **＜0.001** |
| HRS score | 19.58±6.22 | 18.30±7.08 | **＜0.001** |
| pRBD^e^ | 882(42%) | 1006(47.9%) | **＜0.001** |
| RBDQ-HK score | 16.32±16.43 | 18.05±17.11 | **＜0.001** |
| EDS^f^ | 677(32.2%) | 756(36%) | **0.002** |
| ESS score | 7.37±6.16 | 7.94±6.58 | **0.001** |
| Depression^g^ | 685(32.6%) | 654(31.1%) | 0.223 |
| HAMD score | 5.31±5.19 | 5.09±5.44 | **0.014** |
| **Motor complications** |  |  |  |
| Wearing-off^h^ | 757(36%) | 1039(49.5%) | **＜0.001** |
| Dyskinesia^i^ | 288(13.7%) | 369(17.6%) | **＜0.001** |

Data are mean ± SD or n (%), unless otherwise indicated. *P* value are from the paired-sample t-test, Wilcoxon matched-pair signed-rank test, or McNemar–Bowker test. Significant *P* values are indicated in bold. ^a-i^ Evaluated respectively by New Freezing of Gait Questionnaire (NFOGQ), Functional Constipation Diagnostic Criteria Rome Ⅲ (ROME III), MMSE, HRS, RBDQ-HK, ESS, HAMD, 9-item Wearing-off Questionnaire (WOQ-9) and UPDRS part IV-A. PD, Parkinson’s disease; UPDRS, Unified Parkinson’s disease Rating Scale; NMSS, Non-Motor Symptom Scale; PDSS, Parkinson’s Disease Sleep Scale; PDQ-39, The Parkinson's Disease Questionnaire (PDQ-39); SCOPA-AUT, Scales for outcomes in Parkinson’s disease-Autonomic Dysfunction; CI, Cognitive Impairment; MMSE, Mini-Mental State Examination; HRS, Hyposmia Rating Scale; pRBD, probable rapid eye movement sleep behavior disorder; RBDQ-HK, Rapid Eye Movement Sleep Behavior Disorder Questionnaire-Hong Kong; EDS, Excessive daytime sleepiness; ESS, Epworth Sleepiness Scale; HAMD, Hamilton Depression Rating Scale (HAMD-17).

**Supplementary Table 6. Baseline and progression of motor and non-motor outcomes in four different fatigue subgroup****s**

|  | **Never fatigue (Ⅰ=1142)** | |  | **Non-persistent fatigue (Ⅱ=237)** | |  | **New-onset fatigue (Ⅲ=186)** | |  | **Persistent fatigue (Ⅳ=535)** | |
| --- | --- | --- | --- | --- | --- | --- | --- | --- | --- | --- | --- |
| **Outcome** | **Baseline** | **Follow-up** |  | **Baseline** | **Follow-up** |  | **Baseline** | **Follow-up** |  | **Baseline** | **Follow-up** |
| **Motor symptoms** |  |  | |  |  | |  |  | |  |  |
| UPDRS total score | 33.92±17.74 | 36.23±20.64 | | 44.23±19.12 | 41.20±20.18 | | 40.62±21.40 | 51.21±24.85 | | 50.32±21.21 | 56.66±25.41 |
| B (95% CI) [*P* value] |  |  | |  |  | |  |  | |  |  |
| At baseline | 0 [Reference] | | | 9.421 (6.912 to 11.930) [**<0.001**] | | | 5.427 (2.561 to 8.294) [**<0.001**] | | | 12.991 (11.108 to 14.873) [**<0.001**] | |
| For progression | 0 [Reference] | | | -1.094 (-3.569 to 1.382) [0.387] | | | 10.616 (7.713 to 13.520) [**<0.001**] | | | 9.477 (7.286 to 11.668) [**<0.001**] | |
| UPDRS Part III score | 21.69±12.44 | 22.56±14.22 | | 27.95±14.08 | 24.70±13.92 | | 25.96±14.85 | 30.93±16.36 | | 31.02±14.43 | 34.26±16.91 |
| B (95% CI) [*P* value] |  |  | |  |  | |  |  | |  |  |
| At baseline | 0 [Reference] | | | 5.727 (3.863 to 7.591) [**<0.001**] | | | 3.556 (1.493 to 5.620) [**0.001**] | | | 7.319 (5.995 to 8.643) [**<0.001**] | |
| For progression | 0 [Reference] | | | -1.089 (-2.849 to 0.670) [0.225] | | | 5.898 (3.870 to 7.926) [**<0.001**] | | | 6.002 (4.507 to 7.498) [**<0.001**] | |
| Bradykinesia score | 8.02±5.35 | 8.01±5.90 | | 10.29±6.38 | 9.01±5.88 | | 9.65±6.32 | 11.19±6.76 | | 11.44±6.29 | 12.78±7.12 |
| B (95% CI) [*P* value] |  |  | |  |  | |  |  | |  |  |
| At baseline | 0 [Reference] | | | 2.092 (1.236 to 2.947) [**<0.001**] | | | 1.385 (0.481 to 2.289) [**0.003**] | | | 2.753 (2.156 to 3.351) [**<0.001**] | |
| For progression | 0 [Reference] | | | -0.029 (-0.778 to 0.721) [0.940] | | | 2.342 (1.448 to 3.236) [**<0.001**] | | | 2.895 (2.251 to 3.538) [**<0.001**] | |
| Rigidity score | 4.42±3.61 | 4.70±3.95 | | 6.22±4.41 | 5.09±4.02 | | 4.94±4.02 | 6.58±4.52 | | 6.45±4.19 | 7.16±4.72 |
| B (95% CI) [*P* value] |  |  | |  |  | |  |  | |  |  |
| At baseline | 0 [Reference] | | | 1.682 (1.095 to 2.269) [**<0.001**] | | | 0.436 (-0.150 to 1.022) [0.144] | | | 1.767 (1.363 to 2.171) [**<0.001**] | |
| For progression | 0 [Reference] | | | -0.256 (-0.784 to 0.272) [0.342] | | | 1.649 (1.020 to 2.279) [**<0.001**] | | | 1.586 (1.139 to 2.034) [**<0.001**] | |
| Tremor score | 3.06±2.91 | 3.21±3.40 | | 3.59±3.61 | 2.87±3.22 | | 3.83±3.55 | 3.70±3.98 | | 3.63±3.78 | 3.76±3.95 |
| B (95% CI) [*P* value] |  |  | |  |  | |  |  | |  |  |
| At baseline | 0 [Reference] | | | 0.444 (-0.035 to 0.922) [0.069] | | | 0.655 (0.139 to 1.170) [**0.013**] | | | 0.250 (-0.104 to 0.604) [0.166] | |
| For progression | 0 [Reference] | | | -0.617 (-1.028 to -0.207) [**0.003**] | | | 0.093 (-0.437 to 0.622) [0.732] | | | 0.148 (-0.204 to 0.500) [0.410] | |
| Postural instability score | 3.08±2.35 | 3.42±2.88 | | 3.74±2.47 | 4.06±3.09 | | 3.98±2.94 | 4.96±3.47 | | 5.04±3.11 | 5.60±3.73 |
| B (95% CI) [*P* value] |  |  | |  |  | |  |  | |  |  |
| At baseline | 0 [Reference] | | | 0.574 (0.255 to 0.893) [**<0.001**] | | | 0.717 (0.326 to 1.108) [**<0.001**] | | | 1.451 (1.190 to 1.713) [**<0.001**] | |
| For progression | 0 [Reference] | | | 0.266 (-0.111 to 0.642) [0.167] | | | 0.963 (0.536 to 1.390) [**<0.001**] | | | 0.860 (0.553 to 1.167) [**<0.001**] | |
| Freezing of gait^a^ | 249(21.8%) | 347(30.4%) | | 65(27.4%) | 120(50.6%) | | 53(28.5%) | 87(46.8%) | | 221(41.3%) | 284(53.1%) |
| OR (95% CI) [*P* value] |  |  | |  |  | |  |  | |  |  |
| At baseline | 1 [Reference] | | | 1.283 (0.914 to 1.801) [0.150] | | | 1.303 (0.891 to 1.905) [0.172] | | | 2.110 (1.675 to 2.658) [**<0.001**] | |
| For progression | 1 [Reference] | | | 2.308 (1.707 to 3.119) [**<0.001**] | | | 1.885 (1.346 to 2.641) [**<0.001**] | | | 1.940 (1.541 to 2.442) [**<0.001**] | |
| **Nonmotor symptoms** |  |  | |  |  | |  |  | |  |  |
| NMSS total score | 25.86±19.60 | 25.93±22.17 | | 42.27±27.29 | 32.82±23.18 | | 30.77±22.71 | 50.69±32.74 | | 48.68±29.07 | 58.75±35.31 |
| B (95% CI) [*P* value] |  |  | |  |  | |  |  | |  |  |
| At baseline | 0 [Reference] | | | 16.114 (12.453 to 19.775) [**<0.001**] | | | 3.931 (0.695 to 7.167) [**0.017**] | | | 20.022 (17.457 to 22.586) [**<0.001**] | |
| For progression | 0 [Reference] | | | -0.018 (-3.091 to 3.055) [0.991] | | | 21.940 (17.548 to 26.332) [**<0.001**] | | | 21.285 (18.276 to 24.293) [**<0.001**] | |
| PDSS total score | 124.71±30.27 | 123.22±23.41 | | 115.39±24.22 | 118.02±24.62 | | 120.77±21.81 | 111.52±23.06 | | 108.88±25.58 | 107.67±25.03 |
| B (95% CI) [*P* value] |  |  | |  |  | |  |  | |  |  |
| At baseline | 0 [Reference] | | | -9.140 (-12.612 to -5.667) [**<0.001**] | | | -3.033 (-6.524 to 0.457) [0.088] | | | -13.539 (-16.307 to -10.771) [**<0.001**] | |
| For progression | 0 [Reference] | | | -2.971 (-6.305 to 0.364) [0.081] | | | -10.355 (-13.725 to -6.985) [**<0.001**] | | | -10.827 (-13.622 to -8.033) [**<0.001**] | |
| PDQ-39 total score | 19.63±32.55 | 20.09±20.87 | | 33.66±35.77 | 26.33±20.95 | | 24.49±23.74 | 38.37±26.35 | | 37.09±25.09 | 43.65±29.31 |
| B (95% CI) [*P* value] |  |  | |  |  | |  |  | |  |  |
| At baseline | 0 [Reference] | | | 13.248 (8.571 to 17.925) [**<0.001**] | | | 3.403 (-0.241 to 7.048) [0.067] | | | 14.038 (11.075 to 17.001) [**<0.001**] | |
| For progression | 0 [Reference] | | | 2.592 (-1.148 to 6.332) [0.174] | | | 16.093 (12.593 to 19.593) [**<0.001**] | | | 16.882 (13.063 to 20.701) [**<0.001**] | |
| SCOPA-AUT score | 7.05±4.81 | 7.09±5.85 | | 8.89±5.97 | 8.71±6.27 | | 7.93±6.20 | 11.16±7.04 | | 9.93±5.57 | 12.89±7.66 |
| B (95% CI) [*P* value] |  |  | |  |  | |  |  | |  |  |
| At baseline | 0 [Reference] | | | 1.794 (1.005 to 2.583) [**<0.001**] | | | 0.711 (-0.173 to 1.596) [0.115] | | | 2.347 (1.814 to 2.881) [**<0.001**] | |
| For progression | 0 [Reference] | | | 0.972 (0.195 to 1.750) [**0.014**] | | | 3.570 (2.600 to 4.541) [**<0.001**] | | | 4.147 (3.450 to 4.844) [**<0.001**] | |
| Constipation^b^ | 339(29.7%) | 257(22.5%) | | 88(37.1%) | 58(24.5%) | | 66(35.5%) | 72(38.7%) | | 267(49.9%) | 238(44.5%) |
| OR (95% CI) [*P* value] |  |  | |  |  | |  |  | |  |  |
| At baseline | 1 [Reference] | | | 1.431 (1.048 to 1.954) [**0.024**] | | | 1.195 (0.841 to 1.697) [0.320] | | | 1.940 (1.546 to 2.435) [**<0.001**] | |
| For progression | 1 [Reference] | | | 0.996 (0.694 to 1.429) [0.982] | | | 2.233 (1.566 to 3.185) [**<0.001**] | | | 2.124 (1.653 to 2.731) [**<0.001**] | |
| CI^c^ | 54(4.7%) | 88(7.7%) | | 14(5.9%) | 20(8.4%) | | 17(9.1%) | 20(10.8%) | | 64(12.0%) | 92(17.2%) |
| OR (95% CI) [*P* value] |  |  | |  |  | |  |  | |  |  |
| At baseline | 1 [Reference] | | | 1.285 (0.689 to 2.396) [0.431] | | | 1.856 (1.042 to 3.306) [**0.036**] | | | 2.221 (1.500 to 3.289) [**<0.001**] | |
| For progression | 1 [Reference] | | | 1.097 (0.635 to 1.894) [0.740] | | | 1.083 (0.609 to 1.927) [0.785] | | | 1.730 (1.227 to 2.440) [**0.002**] | |
| MMSE score | 27.10±3.05 | 26.95±3.55 | | 26.95±3.07 | 26.88±3.07 | | 26.59±3.26 | 26.46±3.57 | | 25.91±3.97 | 25.49±4.63 |
| B (95% CI) [*P* value] |  |  | |  |  | |  |  | |  |  |
| At baseline | 0 [Reference] | | | -0.197 (-0.606 to 0.212) [0.344] | | | -0.411 (-0.880 to 0.058) [0.086] | | | -0.911 (-1.277 to -0.546) [**<0.001**] | |
| For progression | 0 [Reference] | | | -0.010 (-0.364 to 0.343) [0.954] | | | -0.107 (-0.546 to 0.332) [0.632] | | | -0.515 (-0.856 to -0.174) [**0.003**] | |
| Hyposmia^d^ | 439(38.4%) | 522(45.7%) | | 103(43.5%) | 126(53.2%) | | 74(39.8%) | 101(54.3%) | | 283(52.9%) | 306(57.2%) |
| OR (95% CI) [*P* value] |  |  | |  |  | |  |  | |  |  |
| At baseline | 1 [Reference] | | | 1.245 (0.935 to 1.658) [0.134] | | | 1.028 (0.743 to 1.423) [0.866] | | | 1.638 (1.324 to 2.026) [**<0.001**] | |
| For progression | 1 [Reference] | | | 1.330 (0.937 to 1.887) [0.110] | | | 1.553 (1.072 to 2.251) [**0.020**] | | | 1.221 (0.955 to 1.562) [0.112] | |
| HRS score | 20.23±5.77 | 19.06±6.75 | | 19.27±6.30 | 17.83±7.14 | | 19.54±6.74 | 17.80±7.49 | | 18.33±6.71 | 17.05±7.41 |
| B (95% CI) [*P* value] |  |  | |  |  | |  |  | |  |  |
| At baseline | 0 [Reference] | | | -0.982 (-1.833 to -0.131) [**0.024**] | | | -0.587 (-1.599 to 0.425) [0.256] | | | -1.542 (-2.199 to -0.885) [**<0.001**] | |
| For progression | 0 [Reference] | | | -0.601 (-1.398 to 0.195) [0.139] | | | -0.806 (-1.699 to 0.087) [0.077] | | | -0.675 (-1.286 to -0.065) [**0.030**] | |
| pRBD^e^ | 408(35.7%) | 457(40.0%) | | 109 (46.0%) | 128(54.0%) | | 77(41.4%) | 103(55.4%) | | 288(53.8%) | 318(59.4%) |
| OR (95% CI) [*P* value] |  |  | |  |  | |  |  | |  |  |
| At baseline | 1 [Reference] | | | 1.554 (1.152 to 2.095) [**0.004**] | | | 1.181 (0.843 to 1.654) [0.334] | | | 1.742 (1.401 to 2.165) [**<0.001**] | |
| For progression | 1 [Reference] | | | 1.654 (1.190 to 2.300) [**0.003**] | | | 1.907 (1.320 to 2.755) [**0.001**] | | | 1.630 (1.277 to 2.081) [**<0.001**] | |
| RBDQ-HK score | 13.83±15.45 | 14.98±15.69 | | 18.41±16.82 | 19.05±16.89 | | 15.45±14.94 | 19.95±16.98 | | 21.03±17.64 | 23.49±18.64 |
| B (95% CI) [*P* value] |  |  | |  |  | |  |  | |  |  |
| At baseline | 0 [Reference] | | | 4.399 (2.175 to 6.624) [**<0.001**] | | | 0.931 (-1.278 to 3.140) [0.409] | | | 5.255 (3.543 to 6.967) [**<0.001**] | |
| For progression | 0 [Reference] | | | 1.374 (-0.483 to 3.231) [0.147] | | | 3.801 (1.559 to 6.043) [**0.001**] | | | 3.630 (2.134 to 5.126) [**<0.001**] | |
| EDS^f^ | 282(24.7%) | 297(26.0%) | | 89(37.6%) | 66(27.8%) | | 62(33.3%) | 94(50.5%) | | 244(45.6%) | 299(55.9%) |
| OR (95% CI) [*P* value] |  |  | |  |  | |  |  | |  |  |
| At baseline | 1 [Reference] | | | 1.830 (1.354 to 2.472) [**<0.001**] | | | 1.499 (1.072 to 2.095) [**0.018**] | | | 2.357 (1.886 to 2.945) [**<0.001**] | |
| For progression | 1 [Reference] | | | 0.895 (0.634 to 1.265) [0.531] | | | 2.876 (2.029 to 4.077) [**<0.001**] | | | 2.800 (2.219 to 3.532) [**<0.001**] | |
| ESS score | 6.23±5.63 | 6.38±5.75 | | 8.45±6.71 | 6.42±5.91 | | 7.53±6.36 | 10.47±7.03 | | 9.26±6.39 | 11.06±6.99 |
| B (95% CI) [*P* value] |  |  | |  |  | |  |  | |  |  |
| At baseline | 0 [Reference] | | | 2.140 (1.232 to 3.048) [**<0.001**] | | | 1.207 (0.277 to 2.136) [**0.011**] | | | 2.640 (2.012 to 3.269) [**<0.001**] | |
| For progression | 0 [Reference] | | | -0.819 (-1.583 to -0.054) [**0.036**] | | | 3.514 (2.546 to 4.482) [**<0.001**] | | | 3.203 (2.567 to 3.840) [**<0.001**] | |
| Depression^g^ | 254(22.2%) | 216(18.9%) | | 103(43.5%) | 61(25.7%) | | 60(32.3%) | 86(46.2%) | | 268(50.1%) | 291(54.4%) |
| OR (95% CI) [*P* value] |  |  | |  |  | |  |  | |  |  |
| At baseline | 1 [Reference] | | | 2.660 (1.976 to 3.581) [**<0.001**] | | | 1.595 (1.136 to 2.240) [**0.007**] | | | 3.255 (2.603 to 4.072) [**<0.001**] | |
| For progression | 1 [Reference] | | | 1.109 (0.775 to 1.587) [0.571] | | | 3.508 (2.457 to 5.010) [**<0.001**] | | | 3.832 (3.013 to 4.874) [**<0.001**] | |
| HAMD score | 3.92±4.23 | 3.43±4.24 | | 6.54±5.37 | 4.53±4.22 | | 5.34±5.07 | 7.23±5.75 | | 7.71±5.94 | 8.15±6.44 |
| B (95% CI) [*P* value] |  |  | |  |  | |  |  | |  |  |
| At baseline | 0 [Reference] | | | 2.587 (1.865 to 3.309) [**<0.001**] | | | 1.323 (0.561 to 2.084) [**0.001**] | | | 3.603 (3.046 to 4.160) [**<0.001**] | |
| For progression | 0 [Reference] | | | 0.078 (-0.509 to 0.665) [0.794] | | | 3.184 (2.391 to 3.977) [**<0.001**] | | | 3.137 (2.534 to 3.740) [**<0.001**] | |
| **Motor complications** |  |  | |  |  | |  |  | |  |  |
| Wearing-off^h^ | 330(28.9%) | 462(40.5%) | | 107(45.1%) | 123(51.9%) | | 69(37.1%) | 111(59.7%) | | 251(46.9%) | 343(64.1%) |
| OR (95% CI) [*P* value] |  |  | |  |  | |  |  | |  |  |
| At baseline | 1 [Reference] | | | 1.951 (1.436 to 2.651) [**<0.001**] | | | 1.368 (0.963 to 1.944) [0.080] | | | 1.868 (1.495 to 2.335) [**<0.001**] | |
| For progression | 1 [Reference] | | | 1.288 (0.947 to 1.751) [0.107] | | | 2.114 (1.503 to 2.973) [**<0.001**] | | | 2.255 (1.794 to 2.835) [**<0.001**] | |
| Dyskinesia^i^ | 130(11.4%) | 152(13.3%) | | 42(17.7%) | 46(19.4%) | | 20(10.8%) | 37(19.9%) | | 96(17.9%) | 134(25.0%) |
| OR (95% CI) [*P* value] |  |  | |  |  | |  |  | |  |  |
| At baseline | 1 [Reference] | | | 1.613 (1.073 to 2.425) [**0.022**] | | | 0.669 (0.355 to 1.259) [0.213] | | | 1.399 (1.019 to 1.919) [**0.038**] | |
| For progression | 1 [Reference] | | | 1.335 (0.880 to 2.023) [0.174] | | | 1.724 (1.080 to 2.752) [**0.022**] | | | 1.971 (1.451 to 2.676) [**<0.001**] | |

Data are mean ± SD or n (%), unless otherwise indicated. In each separate generalized estimating equation at baseline, the clinical characteristics at baseline were defined as dependent variables and the analysis was adjusted for age at baseline, sex and disease duration. In each separate generalized estimating equation for progression, the clinical characteristics at follow-up were defined as dependent variables and the analysis was adjusted for age at baseline, sex, disease duration, and baseline values of the clinical factors. Significant *P* values are indicated in bold. ^a-i^ Evaluated respectively by New Freezing of Gait Questionnaire (NFOGQ), Functional Constipation Diagnostic Criteria Rome Ⅲ (ROME III), MMSE, HRS, RBDQ-HK, ESS, HAMD, 9-item Wearing-off Questionnaire (WOQ-9) and UPDRS part IV-A. PD, Parkinson’s disease; UPDRS, Unified Parkinson’s disease Rating Scale; NMSS, Non-Motor Symptom Scale; PDSS, Parkinson’s Disease Sleep Scale; PDQ-39, The Parkinson's Disease Questionnaire (PDQ-39); SCOPA-AUT, Scales for outcomes in Parkinson’s disease-Autonomic Dysfunction; CI, Cognitive Impairment; MMSE, Mini-Mental State Examination; HRS, Hyposmia Rating Scale; pRBD, probable rapid eye movement sleep behavior disorder; RBDQ-HK, Rapid Eye Movement Sleep Behavior Disorder Questionnaire-Hong Kong; EDS, Excessive daytime sleepiness; ESS, Epworth Sleepiness Scale; HAMD, Hamilton Depression Rating Scale (HAMD-17).
